# Proteome-Wide Identification of Lysine Propionylation in the Conidial and Mycelial Stages of *Trichophyton rubrum*

**DOI:** 10.3389/fmicb.2019.02613

**Published:** 2019-11-13

**Authors:** Xingye Xu, Xingwei Cao, Jian Yang, Lihong Chen, Bo Liu, Tao Liu, Qi Jin

**Affiliations:** NHC Key Laboratory of Systems Biology of Pathogens, Institute of Pathogen Biology, Chinese Academy of Medical Sciences & Peking Union Medical College, Beijing, China

**Keywords:** dermatophytes, *Trichophyton rubrum* (*T. rubrum*), posttranslational modifications (PTMs), lysine propionylation, proteome

## Abstract

Posttranslational modifications (PTMs) exist in a wide variety of organisms and play key roles in regulating various essential biological processes. Lysine propionylation is a newly discovered PTM that has rarely been identified in fungi. *Trichophyton rubrum* (*T. rubrum*) is one of the most common fungal pathogens in the world and has been studied as an important model organism of anthropic pathogenic filamentous fungi. In this study, we performed a proteome-wide propionylation analysis in the conidial and mycelial stages of *T. rubrum*. A total of 157 propionylated sites on 115 proteins were identified, and the high confidence of propionylation identification was validated by parallel reaction monitoring (PRM) assay. The results show that the propionylated proteins were mostly involved in various metabolic pathways. Histones and 15 pathogenicity-related proteins were also targets for propionylation modification, suggesting their roles in epigenetic regulation and pathogenicity. A comparison of the conidial and mycelial stages revealed that most propionylated proteins and sites were growth-stage specific and independent of protein abundance. Based on the function classifications, the propionylated proteins had a similar distribution in both stages; however, some differences were also identified. Furthermore, our results show that the concentration of propionyl-CoA had a significant influence on the propionylation level. In addition to the acetylation, succinylation and propionylation identified in *T. rubrum*, 26 other PTMs were also found to exist in this fungus. Overall, our study provides the first global propionylation profile of a pathogenic fungus. These results would be a foundation for further research on the regulation mechanism of propionylation in *T. rubrum*, which will enhance our understanding of the physiological features of *T. rubrum* and provide some clues for the exploration of improved therapies to treat this medically important fungus.

## Introduction

Posttranslational modifications (PTMs) are widely present in many kinds of organisms, giving rise to many potential “proteoforms” (Compton et al., 2018). In particular, reversible PTMs can efficiently control protein function and biological processes without expending energy to modulate the transcription and translation that regulate these processes (Bontemps-Gallo et al., 2018). Among these PTMs, lysine acetylation is one of the most common PTMs and has been studied in many organisms (Liu F. et al., 2014; Zhou et al., 2016). In addition to acetylation, many other types of lysine acylation have also been discovered, including malonylation, glutarylation, succinylation, methylation, propionylation and butyrylation (Zhang et al., 2009; Hirschey and Zhao, 2015; Cheng et al., 2019).

Posttranslational modifications are involved in various complex regulatory mechanisms of cellular processes and have shown their physiological significance in cell growth, microbial evolution, stress and environment adaptation (Okanishi et al., 2017). In addition, PTMs also function in the regulation of pathogenesis (Leach and Brown, 2012). For instance, in *Salmonella typhimurium*, the acetylation of HilD at K297 reduces the capacity of *S. typhimurium* to invade HeLa cells and attenuates virulence in a mouse model, indicating that deacetylation of HilD at this site is crucial for virulence (Sang et al., 2017). In *Aspergillus flavus*, the succinylation site at K370 of AflE is crucial for the production of aflatoxin, which can cause chronic toxicity, liver cancer and even death in both human and animals (Ren et al., 2018).

Dermatophytes are one of the most widespread pathogenic fungi, and the infections caused by these fungi have recently increased, with an estimated prevalence of 20–25% worldwide (Song et al., 2018). Infections caused by dermatophytes are not limited to superficial mycosis, and cases of deep dermatophytosis have also been reported (Lanternier et al., 2013). Dermatophyte infections have gained attention due to their significant social, health, and economic implications, which constitute an important public health problem (Ouf et al., 2016; Rezaei-Matehkolaei et al., 2018). Despite the availability of many classes of antifungal agents that combat dermatophytes, the prevalence of these fungi remains unchanged (Rudramurthy et al., 2018). Conventional antifungal drugs often have adverse effects, and the treatment period is typically long (Sit et al., 2018). In addition, resistance to antifungal drugs has been reported, and recurrence occurs frequently (Gaziano et al., 2018; Sit et al., 2018).

*Trichophyton rubrum* (*T. rubrum*) is the leading pathogen accounting for 69.5% of all cases of dermatophytosis and is considered an important model species to study anthropic pathogenic filamentous fungi (Liu et al., 2007; Petrucelli et al., 2018). *T. rubrum* has two major growth stages that alternate between a unicellular conidial form and a multicellular mycelial form (Liu et al., 2007). This transition in growth form is dependent on environmental conditions (Liu et al., 2007). Conidia are dormant or quiescent structures that are mainly responsible for transmission and defense against adverse conditions (Li et al., 2017). In addition, studies have shown that conidia also play a role in host adhesion and infection (Wang Z. et al., 2018). After the inoculation of conidia into the host stratum corneum, mycelia are formed, which take advantage of the nutrients in skin tissue and grow vigorously to aggravate skin damage (Liu et al., 2007). An understanding of the features of *T. rubrum* in each growth stage would facilitate further investigation of the developmental and physiological properties of this fungus, which could provide a foundation for the identification of enhanced therapies to treat this medically important fungus.

In recent years, proteome-wide analyses of acetylation and succinylation modifications have been performed in *T. rubrum* conidial and mycelial stage respectively, which has greatly improved our understanding of the PTMs in this fungus (Xu X. et al., 2017; Xu et al., 2018b). Kpr is a newly discovered PTM (Chen et al., 2007). Some studies have shown the significant roles of Kpr in epigenetic regulation and cellular stress responses in both eukaryotes and bacteria (Okanishi et al., 2014). For example, the mitochondrial protein propionylation level increased in the mouse liver during ethanol-diet feeding, indicating that propionylation may play a role in the response to stress (Fritz et al., 2013). Based on an RNA-seq assay of the mouse liver, propionylated histone site H3K14 (H3K14pr) was shown to be associated with transcriptional activation, and lipid metabolism pathways were suggested to be the primary targets of H3K14pr in fasted mice (Kebede et al., 2017). Although investigations of Kpr have been performed in some organisms, the current understanding of the features of this kind of PTM, including its functions, roles and cellular distributions, is insufficient. Especially in fungi, global Kpr identification has not been well reported.

In the present study, we performed the first lysine propionylome analysis in *T. rubrum*, which is also the first proteome-wide propionylation identification in pathogenic fungi. Kpr differs from lysine acetylation by only one carbon atom (Sabari et al., 2017). Due to the similarity of the chemical structures of these two types of lysine residues, we questioned whether lysine acetylation and propionylation were similarly distributed in the *T. rubrum* proteome. However, the results show that Kpr is much less abundant than lysine acetylation. Although rare in *T. rubrum*, the important roles of propionylation were revealed with further bioinformatics analysis.

## Materials and Methods

### Strains and Culture

*Trichophyton rubrum* strain BMU 01672 was cultured on potato dextrose agar (BD, Sparks, MD, United States) at 28°C to produce conidia. The conidia were harvested with distilled water on ice and filtered through Miracloth (Merck, Billerica, MA, United States) and a 400 and 600 mesh sieve sequentially. The conidia purity was examined with a microscope. The mycelia were cultured in Sabouraud liquid medium (BD) at 28°C with constant shaking (180 rpm). The mycelia were harvested by washing the cultures with distilled water to remove the medium.

### Protein Extraction

The fungal sample was first ground in liquid nitrogen, resolved in lysis buffer (8 M urea, 10 mM dithiothreitol (DTT), 50 mM nicotinamide (NAM), 3 μM trichostatin A (TSA) and 0.1% protease inhibitor cocktail) and sonicated on ice three times. After centrifugation at 20,000 *g* at 4°C, the supernatant was collected. Finally, proteins were precipitated with 15% trichloroacetic acid (TCA) at −20°C, and the precipitate was washed with cold acetone two times. The proteins were redissolved in 8 M urea (containing 100 mM NH_4_HCO_3_, pH 8.0) and quantified using the 2-D Quant Kit (GE Healthcare, Piscataway, NJ, United States).

### Trypsin Digestion

The proteins were reduced with 10 mM DTT and alkylated with 20 mM iodoacetamide (IAA). Then, the proteins were digested with trypsin (Promega, Madison, WI, United States) at a trypsin/protein ratio of 1:50 (w/w).

### High-Performance Liquid Chromatography (HPLC) Fractionation

The peptides were fractionated by high pH reverse-phase HPLC using an Agilent 300Extend C18 column (4.6 mm × 250 mm, 5 μm, 300A°, Agilent Technologies, Santa Clara, CA, United States) with a gradient of 2–60% acetonitrile (containing 10 mM ammonium bicarbonate, pH 10) over 80 min into 80 fractions. Then, the peptides were combined into 6 fractions for each conidial and mycelial sample according to the method described previously, and the fractions were dried completely (Xu et al., 2018b).

### Affinity Enrichment of Propionylated Peptides

The peptides were redissolved in NETN buffer (100 mM NaCl, 1 mM EDTA, 50 mM Tris-HCl, and 0.5% NP-40, pH 8.0) and then incubated with preconjugated pan anti-propionyl-lysine agarose antibody beads (PTM Biolabs, Hangzhou, Zhejiang, China) overnight at 4°C. The beads were washed with NETN buffer and ddH_2_O subsequently. The bound peptides were eluted with 0.1% trifluoroacetic acid (TFA) and then cleaned with C18 ZipTips (Millipore, Billerica, MA, United States).

### Liquid Chromatography-Tandem Mass Spectrometry (LC-MS/MS) Analysis

The peptides were dissolved in 0.1% formic acid (FA), loaded onto an Acclaim PepMap 100 reverse-phase C18 precolumn (75 μm × 2 cm, 3 μm, 100A°, Thermo Fisher Scientific, Waltham, MA, United States) and separated on an Acclaim PepMap RSLC reverse-phase C18 analytical column (75 μm × 15 cm, 3 μm, 100A°, Thermo Fisher Scientific) using the EASY-nLC 1000 UPLC system (Thermo Fisher Scientific). The gradient was set as 7–25% solvent B (0.1% FA in 98% ACN) for 24 min, 25–40% B for 8 min, increased to 80% B in 5 min and was then held at 80% B for 3 min, all at a constant flow rate of 350 nl/min.

The eluted peptides were directly subjected to an NSI source followed by tandem mass spectrometry (MS/MS) analysis in Q Exactive Plus (Thermo Fisher Scientific) coupled online to the UPLC. For MS scans, the m/z scan range was set as 350–1800. Peptides were detected in the orbitrap at a resolution of 70,000 and were selected for MS/MS at 28% normalized collision energy (NCE) using higher energy collision dissociation (HCD). The fragment ions were detected in the orbitrap at a resolution of 17,500. A DDA procedure was applied that alternated between one MS scan followed by 20 MS/MS scans, with a threshold of ion count above 1E^4^ in the MS scan and with 10.0 s dynamic exclusion. Three biological replicates were performed.

### Database Searches

The raw data of the MS/MS results were processed using MaxQuant software with an integrated Andromeda search engine (v.1.5.2.8) (Cox and Mann, 2008). The mass spectra were searched against the *T. rubrum* protein database version 2 downloaded from the website of BroadInstitute^1^ (containing 10,418 sequences, commonly observed contaminants were appended to the database) and concatenated with a reverse decoy database. The false-discovery rate (FDR) was calculated using this target-decoy search strategy.

Trypsin was specified as the enzyme; up to 4 missed cleavages were allowed; up to 5 modifications and 5 charges per peptide were permitted; a mass error of 10 ppm was set for precursor ions and 0.02 Da for fragment ions; carbamidomethylation on Cys was set as fixed modification; and oxidation on Met, propionylation on Lys and acetylation on the N-terminus were specified as dynamic modifications. FDR < 1% was specified for the protein, peptide and modification sites. The minimum peptide length was 7, and the peptide score was more than 40. The site localization probability was set as ≥0.75.

### Bioinformatics Analysis

GO annotations of the proteome were derived from the UniProt-GOA database^2^ and assisted with InterProScan software to annotate the protein’s GO function based on protein sequence alignment (Zdobnov and Apweiler, 2001). WoLF PSORT was used to predict subcellular localization (Horton et al., 2007). The KEGG database was used to annotate the protein pathway using KEGG online service tools KAAS and KEGG mapper. Motif-X was used to analyze amino acid sequence models surrounding the propionylated lysines.

### Parallel Reaction Monitoring (PRM) Analysis of the Synthetic Peptides

To validate the MS/MS results of the propionylation identification, two propionylated peptides, AISLDK(pro) THGISAR and LLIQNQDEMLK(pro)SGR, were synthesized by the Chinese Peptide Company (Hangzhou, Zhejiang, China) and detected by the PRM approach. Four nanograms of the synthetic peptide sample was loaded onto the UPLC system with the identical gradient and flow rate setting in the DDA mode for propionylated peptide identification. Then, the eluate was analyzed via MS using Q Exactive^TM^ Plus (Thermo Fisher Scientific) in DIA PRM mode. A full mass spectrum was detected at a resolution of 70,000, and the m/z range was 350–1000. The MS/MS scans were triggered by inclusion list (Supplementary Table S1) and fragmented using HCD at a 28% NCE. The resolution of the MS/MS scans was 17,500, the AGC target was 5E^4^ and the maximum injection time was 200 ms.

The raw files of the propionylome assay acquired in DDA mode were imported into the Skyline (v.3.6) software to create a spectral library (MacLean et al., 2010). The precursor and fragment ions (typically b- and y-ions) identified in the PRM assay were matched to specific peptides present in the spectral libraries with the following parameters: enzyme was set as trypsin [KR/P]; max missed cleavage was 0; peptide length was 7–25; fixed modification was carbamidomethylation on Cys; and dynamic modifications were oxidation on Met and propionylation on Lys. Transition settings were set as follows: precursor charges were 2 and 3; ion charges were 1; ion types were b and y; product ion selection was from ion 3 to the last ion; and ion match tolerance was 0.02 Da. Finally, the ‘dotp’ value was calculated, which is a dot-product similarity metric between the measured PRM peak areas and the MS/MS library peak intensities. As demonstrated by the Skyline website^3^, a ‘dotp’ value closer to 1.0 indicates a better the match.

### Western Blot Analysis

The *T. rubrum* conidial and mycelial proteins were extracted as described above. Briefly, cells were ground in liquid nitrogen, and the powder was suspended in lysis buffer. Proteins were precipitated with 15% TCA and then quantified. The proteins were separated on 12% sodium dodecyl sulfate polyacrylamide gel electrophoresis (SDS-PAGE) and then transferred to a polyvinylidene fluoride (PVDF) membrane (Invitrogen, Carlsbad, CA, United States). The membrane was blocked with 2% bovine serum albumin (BSA, MP Biomedicals, Illkirch Cedex, France) in Tris-buffered saline solution containing 0.05% Tween 20 (TBST) at room temperature for 1 h and then incubated with primary antibody, including pan anti-propionylation (K), anti-butyrylation (K), anti-dimethylation (K), anti-trimethylation (K), anti-2-hydroxyiso-butyrylation (K), anti-malonylation (K), anti-ubiquitylation (K) and anti-glutarylation (K) antibodies (PTM Biolabs) at 4°C overnight. The membrane was washed with TBST for 5 min three times and then incubated with (HRP)-conjugated goat anti-rabbit antibody (Pierce, Dallas, TX, United States) for 1 h. After the membranes were washed three times with TBST, the bands were visualized using SuperSignal West Pico PLUS Chemiluminescent Substrate (Thermo, Rockford, IL, United States).

### Detection of the Intracellular Propionyl-CoA Concentration

The *T. rubrum* samples were obtained as described above, and metabolism was quenched in liquid nitrogen. About 50 mg of each sample was transferred into a 2 ml centrifuge tube, then 800 μl methanol was added and vortexed for 1 min. Samples were further grinded at 60 Hz for 90 s, ultrasonic treated for 30 min at 4°C, and then placed at −20°C for 1 h. After centrifugation at 12000 rpm for 15 min at 4°C, 200 μl supernatant for each sample was obtained and used for propionyl-CoA concentration examination. The standard propionyl-CoA was purchased from Sigma (St. Louis, MO, United States), and diluted to the concentration of 500, 200, 100, 50, 20, and 10 ng/ml respectively.

The samples were separated using ACQUITY UPLC system (Waters, Milford, MA, United States) equipped with ACQUITY UPLC BEH T3 column (100 × 2.1 mm, 1.7 μm, Waters). The sample injection volume was 10 μl; the flow rate was constant at 0.35 ml/min and column temperature was maintained at 40°C. The mobile phase consisted of 0.1% formic acid and 5 mM ammonium formate in water (solvent A), and 100% acetonitrile (solvent B). The gradient was set as 0–0.5 min: 90% B; 0.5–1 min: 90%–30% B; 1–2 min: 30%–5% B; 2–4 min: 5% B; 4–4.01 min: 5%–90% B; and 4.01–5 min: 90% B for each sample.

Samples were detected using API5500 tandem mass spectrometer (Sciex, Framingham, MA, United States) by multiple reaction monitoring (MRM) in negative ion mode. The parameters for MS were set as follows: collision gas: 7 arb; curtain gas: 35 arb; ion spray voltage: 4500 V; temperature: 550°C; ion source gas 1: 55 arb and ion source gas 2: 55 arb. The MRM transitions were m/z 822/408 for quantitative analysis and 822/426 for qualitative analysis. The retention time for propionyl-CoA detection was 1.62 min. Three biological replicates were performed for each sample.

The standard curve was determined by detection of standard propionyl-CoA with distinct concentration (Supplementary Figure S1). The total ion current (TIC) and the retention time for each sample are shown in Supplementary Figure S2.

### Fungal Viability Assay

The *T. rubrum* (1 to 3 × 10^5^ CFU/mL) were cultured in Sabouraud liquid medium and Sabouraud supplemented with different concentrations of sodium propionate (0.125, 0.25, 0.5, and 1% sequentially) at 28°C for 7 days. XTT cell proliferations assay kit (Abnova, Taipei, Taiwan) was used to exam the fungal viability according to the manufacturer’s instruction. Briefly, every 100 μl culture in 96-well microplates was added by 10 μl of XTT solution and incubated for 3 h. The resulting absorbance of each sample was read at a wavelength of 450 nm using an infinite M200 pro microplate reader (Tecan, Mannedorf, Switzerland). Three replicated experiments were performed.

### Identification of the 26 PTM Types in *T. rubrum*

*Trichophyton rubrum CBS 118892* protein sequences, genome sequences and transcript sequences were downloaded from the NCBI GenBank database^4^. The raw proteome data were downloaded from Peptide Atlas with the dataset identifier PASS01111 (Xu et al., 2018a). We performed the combined unrestrictive searching strategy using MODa (version 1.23) (Na et al., 2012) and the specific PTM searching approach using MaxQuant (version 1.6.0.16) (Cox and Mann, 2008) as previously described (Yang et al., 2018). A total of 26 PTMs were selected to search for their possible presence in *T. rubrum*. The detailed parameters used in database searching are listed in Supplementary Table S2A.

## Results

### Proteome-Wide Identification of Lysine Propionylation in *T. rubrum*

**FIGURE 1:**
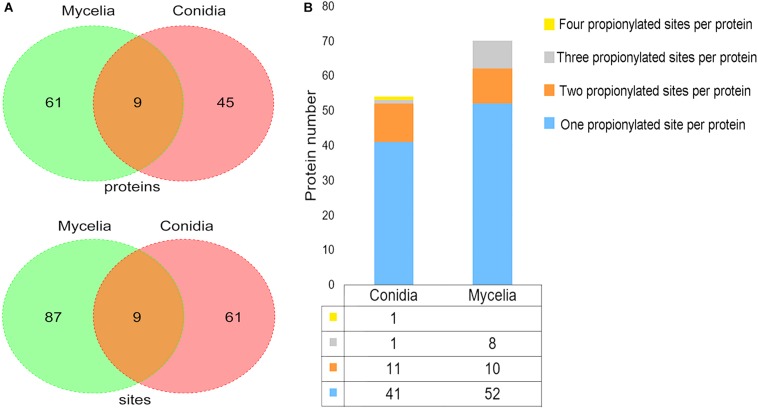
The propionylated proteins and sites identified in *T. rubrum*. **(A)** The propionylated proteins and sites identified in the conidial and mycelial stages respectively. **(B)** The propionylated sites per protein identified in the conidial and mycelial stages respectively.

To identify the low-abundance propionylated peptides, we used an anti-propionyl-lysine antibody to enrich propionylated peptides before LC-MS/MS analysis. As a result, 157 propionylated sites on 115 proteins were identified in both stages based on three biological replicates (Supplementary Table S3). The propionylation analysis was performed separately in the conidial and mycelial stages. A total of 70 propionylated sites on 54 proteins were identified in conidia, and 96 propionylated sites on 70 proteins were identified in mycelia (Figure 1A). The propionylated sites per protein were slightly more abundant in mycelia (1.37) than in conidia (1.30), and the majority of proteins had one propionylated site in both stages (Figure 1B). Although the propionylation level was similar in conidia and mycelia, most propionylated sites and proteins were growth-stage specific. As shown in Figure 1A, 61 propionylated sites and 45 propionylated proteins were specific to conidia, and 87 propionylated sites and 61 propionylated proteins were specific to mycelia.

### PRM Validation of the Propionylated Peptide Identification

Due to the specific amino composition, each peptide possess the specific retention time and MS/MS spectrum in the LC-MS/MS identification. Two propionylated peptides were synthesized and identified by LC-MS/MS in PRM mode. The retention time and the MS/MS spectrum were compared between the synthetic peptides identified in PRM mode and the corresponding peptides identified in the initial DDA mode, allowed us to validate the propionylation identified in our study. As shown in Supplementary Table S1, the two synthetic peptides displayed the similar retention time in the PRM mode and their corresponding peptides identified in DDA mode. In addition, the precursor and fragment ions of synthetic peptides were compared to the specific peptides identified in the DDA mode to compare the similarity of the fragmentation pattern and the ion intensity. The similarity was measured by calculating the ‘dotp’ value, and a ‘dotp’ value closer to 1.0 indicates a better match. As shown in Supplementary Figure S3, for the peptide 1 (AISLDK(pro)THGISAR), the ‘dotp’ values were 0.92 for +2 charged ions and 0.95 for +3 charged ions respectively. As for peptide 2 (LLIQNQDEMLK(pro)SGR), the ‘dotp’ value was 0.95 for the +2 charged ions (no +3 charged ions were identified in the initial DDA identification, thus there is no ‘dotp’ values for this ion type). These results suggest the high confidence of the propionylation identification in our study.

### Sequence Context of the Propionylated Sites

**FIGURE 2:**
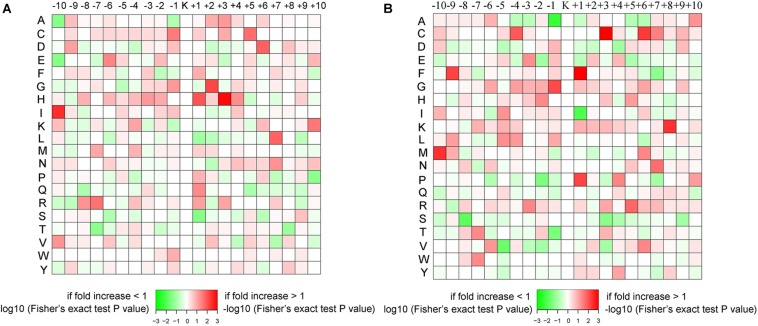
The amino acid sequence pattern surrounding the propionylated lysines. The colors represent the enrichment (red) or depletion (green) of amino acids at the specific positions. **(A)** Conidial stage. **(B)** Mycelial stage.

The amino sequence surrounding the propionylated lysines (Kpr) may indicate the preference of enzymes that catalyze the propionylation modification. In *Thermus thermophilus*, acidic amino acids have been suggested to occupy the +1 position of Kpr preferentially (Okanishi et al., 2014). However, in our study, as shown in Figure 2, the acidic amino acids aspartic acid (D) and glutamic acid (E) do not show this sequence pattern. In the conidial stage, D and E show a slight preference for the −1 and −2 positions, respectively. In the mycelial stage, D and E were enriched at the – 1 position and depleted at the +1 position. The basic amino acids [lysine (K), arginine (R) and histidine (H)] were slightly enriched in the general vicinity of Kpr (from −10 to +10 positions) in the mycelial stage. In the conidial stage, K and R were not significantly enriched surrounding Kpr, while only H showed a preference in the vicinity of Kpr and was especially significantly enriched at the +1 and +3 positions. In addition, a number of hydrophobic and hydrophilic amino acids showed different characteristics between the conidial and mycelial stages. For instance, in conidia, the hydrophobic amino acids alanine (A) and proline (P) were depleted at the −10 and +10 positions, respectively, and isoleucine (I) and glycine (G) were greatly enriched at the −10 and +2 positions, respectively. The hydrophilic amino acid serine (S) was significantly depleted at the +1 position, and threonine (T) was significantly depleted at both the −7 and +8 positions. In mycelia, the hydrophobic amino acid P was significantly depleted at the −2 position and enriched at the +1 position, phenylalanine (F) was significantly enriched at the −9 and +1 positions, and the hydrophilic amino acid cysteine (C) was significantly enriched at the −4, +3, and +6 positions. These differences in sequence patterns between the two stages may suggest that the enzymes that regulate propionylation may recognize specific substance sequences in each stage.

### Gene Ontology (GO) and Subcellular Localization Classification of Propionylated Proteins

**FIGURE 3:**
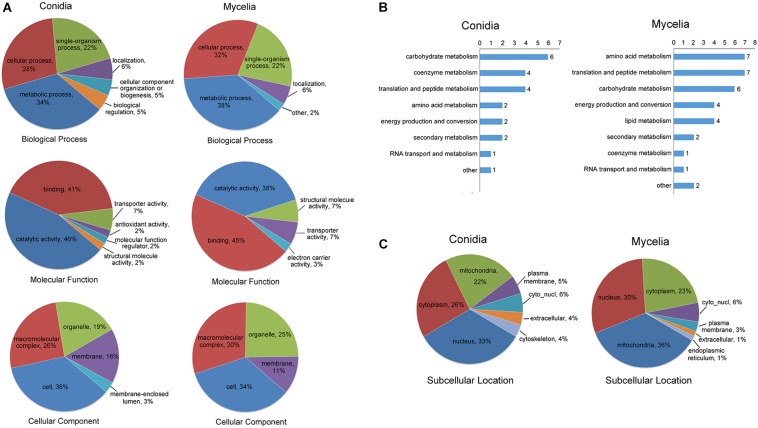
Classification of the propionylated proteins based on GO and subcellular location for the conidial and mycelial stages, respectively. **(A)** GO classification of the propionylated proteins. **(B)** Classification of the propionylated proteins involved in metabolism. **(C)** Subcellular location classification of the propionylated proteins.

We performed GO classification for all propionylated proteins in the conidial and mycelial stages (Figure 3A and Supplementary Table S4). Based on the biological process classification, metabolic process was the largest category for propionylated proteins in both stages (34% in conidia and 38% in mycelia), followed by cellular process (28% in conidia and 32% in mycelia) and single-organism process (22% in both conidia and mycelia). Based on molecular function classification, the percentage of binding increased from 41% in conidia to 45% in mycelia, and the percentage of catalytic activity decreased from 46% in conidia to 38% in mycelia. Based on the cellular component classification, the cell (36% in conidia and 34% in mycelia), macromolecular complex (26% in conidia and 30% in mycelia), organelle (19% in conidia and 25% in mycelia) and membrane (16% in conidia and 11% in mycelia) were the four major categories in both stages.

As described above, metabolism is the largest category in both stages, and the number of propionylated proteins in this category increased from 22 in conidia to 34 in mycelia. To further investigate the metabolic processes in which propionylated proteins are most involved, the metabolism category was classified into subcategorization. As shown in Figure 3B, the subclasses of metabolism include carbohydrate metabolism, amino acid metabolism, and coenzyme metabolism. These subclasses of propionylated proteins were similar between the conidial and mycelial stages, except for lipid metabolism, which was found only in the mycelial stage. In addition, the number of proteins related to coenzyme metabolism decreased from 4 in conidia to 1 in mycelia, and proteins involved in amino acid metabolism increased from 2 in conidia to 7 in mycelia.

The subcellular localization analysis indicated that the propionylated proteins exhibited significant differences in the conidial and mycelial stages (Figure 3C and Supplementary Table S5). In conidia, the largest category was nucleus (33%), followed by cytoplasm (26%) and mitochondria (22%). In mycelia, the three largest categories were mitochondria (36%), nucleus (30%) and cytoplasm (23%). In addition, the propionylated proteins in the extracellular matrix and cytoskeleton were also more abundant in conidia than in mycelia.

### Kyoto Encyclopedia of Genes and Genomes (KEGG) Enrichment Analysis of Propionylated Proteins

**FIGURE 4:**
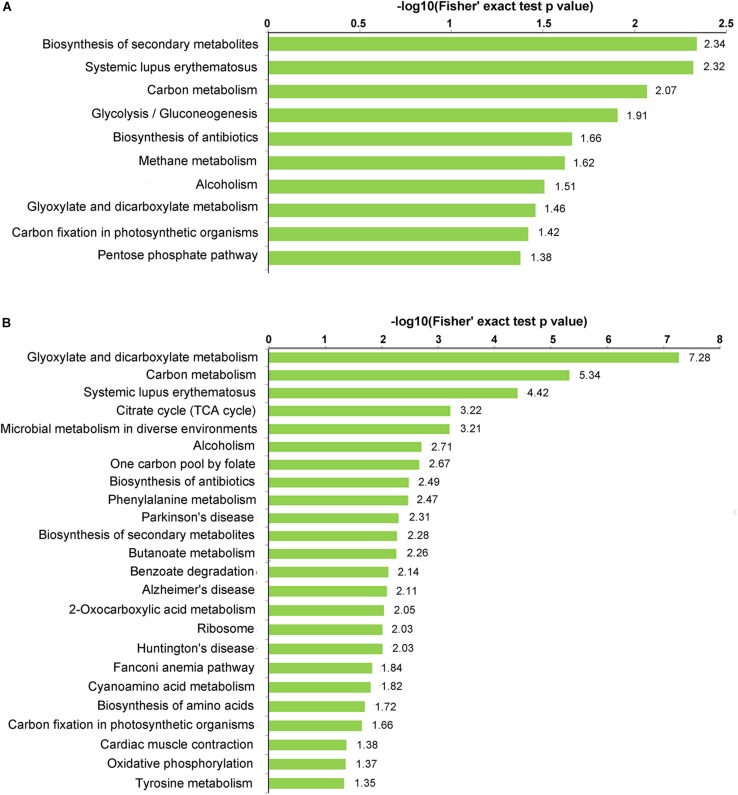
KEGG enrichment of the propionylated proteins. **(A)** Conidial stage. **(B)** Mycelial stage.

KEGG enrichment analysis was performed to investigate which pathways were most enriched with propionylated proteins (Figure 4 and Supplementary Table S6). Secondary metabolites (SMs) have been suggested to play essential roles in the interactions between fungus and host and are involved in fungal infection and pathogenicity (Sbaraini et al., 2016; Pfannenstiel et al., 2018; Wang R. et al., 2018). Based on the KEGG enrichment analysis, the propionylated proteins were involved in secondary metabolism, including the biosynthesis of SMs and the biosynthesis of antibiotics, which were enriched in both stages, and microbial metabolism in diverse environments, which was significantly enriched in mycelia. In addition, the pathways including systemic lupus erythematosus, carbon metabolism, alcoholism, glyoxylate and dicarboxylate metabolism, and carbon fixation in photosynthetic organisms were enriched in both stages. Additionally, some pathways were differentially enriched in the two stages, including glycolysis/gluconeogenesis, methane metabolism, and the pentose phosphate pathway, which were significantly enriched in conidia, and the citrate cycle, ribosome, biosynthesis of amino acids, and oxidative phosphorylation pathways, which were enriched in mycelia.

### Propionylated Proteins Related to Fungal Pathogenicity

Of the propionylated proteins identified in our study, some were reported to be related to fungal pathogenicity. These proteins are listed in Table 1. C-14 sterol reductase (TERG_00475T1) was found to be propionylated at site K338. Knocking out the C-14 sterol reductase in *Candida albicans* caused severe defects in hyphal growth, which is a major contribution to fungal pathogenicity (Luna-Tapia et al., 2015). A similar effect was detected with the cyclin-dependent kinase Pho85 (TERG_05145T3, K663), which is required for polar growth to establish an infective filament, which thus contributes to fungal virulence (Castillo-Lluva et al., 2007). The protein peroxisomal multifunctional beta-oxidation protein MFP (TERG_05621T0, K475) has also been suggested to play roles in sporulation, filamentous growth and virulence in *Ustilago maydis* (Kretschmer et al., 2012). Additionally, defects in peroxisome biogenesis and β-oxidation prevent appressorium-based infection in *Colletotrichum lagenarium*, *Magnaporthe grisea*, and *Alternaria alternata* (Kretschmer et al., 2012).

**TABLE 1:** The identified propionylated proteins related to fungal pathogenicity.

				**Modified**
**Protein**		**Amino**		**with Kac^a^**
**accession**	**Position**	**acid**	**Protein description**	**and Ksucc^b^**
TERG_00066T0	105	K	4-hydroxyphenylpyruvate dioxygenase	Kac, Ksucc
TERG_00475T1	338	K	C-14 sterol reductase	
TERG_01573T0	120	K	Peptidyl-prolyl cis-trans isomerase	Kac, Ksucc
TERG_06655T0	105	K	Peptidyl-prolyl cis-trans isomerase	Kac
TERG_06858T0	88	K	Peptidyl-prolyl cis-trans isomerase	Kac, Ksucc
TERG_02242T0	70	K	Adhesin, putative	Kac
TERG_02508T0	1559	K	ATP-binding cassette transporter	
TERG_03357T0	1000	K	Non-ribosomal peptide synthase	
TERG_04281T2	168	K	Woronin body protein HexA	Kac, Ksucc
TERG_04335T0	198	K	Superoxide dismutase [Cu-Zn]	
TERG_03206T0	119	K	Heat shock protein 70 (Hsp70)	
TERG_03206T1	58	K	Heat shock protein 70 (Hsp70)	Kac, Ksucc
TERG_06505T0	511	K	Molecular chaperone Hsp70	Kac, Ksucc
TERG_05145T3	663	K	Cyclin dependent kinase (Pho85)	
TERG_05621T0	475	K	Peroxisomal multifunctional beta-oxidation protein (MFP)	Kac

*^*a*^Kac, lysine acetylation; ^*b*^Ksucc, lysine succinylation.*

Peptidyl-prolyl *cis/trans* isomerases (PPIases) are rate-limiting enzymes in the protein folding process (Unal and Steinert, 2014). Growing evidence has shown the virulence-associated functions of this protein family and their potential as drug targets in pathogens (Unal and Steinert, 2014). In our study, three propionylated PPIases, TERG_01573T0 (K120), TERG_06655T0 (K105) and TERG_06858T0 (K88), were identified. Another propionylated protein, 4-hydroxyphenylpyruvate dioxygenase hpdA (TERG_00066T0, K105), which catalyzes the second step of tyrosine catabolism, has also been suggested to play a role in the pathogenicity of *Penicillium marneffei* (Boyce et al., 2015).

Adhesion, which mediates the pathogen and host interaction, is involved in the first step of infection and is a major virulence factor contributing to fungal pathogenicity (Timmermans et al., 2018). One adhesin (TERG_02242T0) was found to be propionylated at the K70 site. Furthermore, non-ribosomal peptide synthetases (NRPSs) are a class of enzymes that catalyze the biosynthesis of small bioactive peptides independent of ribosomes (Condon et al., 2013). NRPSs contribute to the biosynthesis of virulence determinants and SMs, which play essential roles in fungal pathogenicity (Donzelli et al., 2017). In our study, the NRPS TERG_03357T0 was identified to be propionylated at K1000.

Superoxide dismutase and heat shock proteins (HSPs) are involved in virulence-associated stress responses (Liu et al., 2018). In our study, a total of one superoxide dismutase [Cu-Zn] TERG_04335T0 (K198) and three HSP proteins, TERG_03206T0 (K119), TERG_03206T1 (K58) and TERG_06505T0 (K511), were identified as propionylated proteins. The woronin body protein HexA (TERG_04281T2, K168) has also been shown to be important for stress resistance and virulence in the plant pathogen *Fusarium graminearum* and the human opportunistic pathogen *Aspergillus fumigatus* (Liang et al., 2017). In addition, ATP-binding cassette transporters (ABC transporters, TERG_02508T0, K1559), which transport drugs outside cells using energy derived from ATP hydrolysis, are also important virulence factors in pathogenic fungi (Xie et al., 2016).

When comparing these propionylated proteins with the previously identified proteins with other PTMs in *T. rubrum*, we found that 9 proteins were commonly modified by acetylation and/or succinylation (Xu X. et al., 2017; Xu et al., 2018b). Whether propionylation play a role in regulating fungal pathogenicity and the cooperation between propionylation and other PTMs involved in pathogenicity-related pathways require further investigation.

### Propionylation Modification on Histones

**FIGURE 5:**
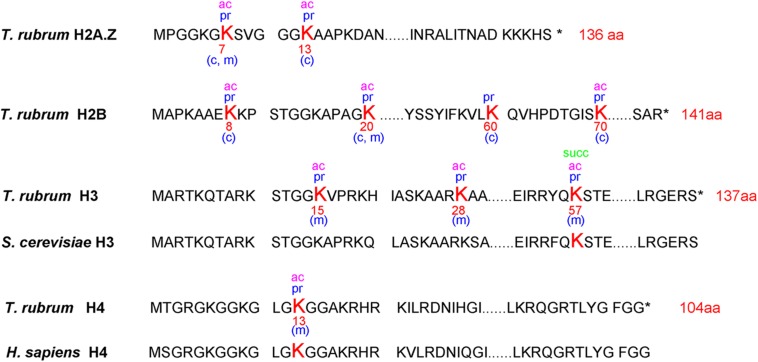
The identified propionylated sites on histones and those conserved in other species. The lysine (K) shown in bold red font represents the propionylated site. The letter “c” in brackets indicates that the site is modified in the conidial stage, and the letter “m” in brackets indicates that the site is modified in the mycelial stage.

A total of 10 propionylated sites were identified on histones (Figure 5). Only H2A. Z (Lys-7) and H2B (Lys-20) were commonly propionylated at both stages. H2B (Lys-8), H2B (Lys-60), H2B (Lys-70) and H2A. Z (Lys-13) were specific to the conidial stage, and H3 (Lys-15), H3 (Lys-28), H3 (Lys-57) and H4 (Lys-13) were specific to the mycelial stage. PTMs on histones have been suggested to participate in epigenetic regulation (Nardelli et al., 2013). These differences in histone propionylation between the conidia and mycelia stages indicate that propionylation may be differentially involved in epigenetic gene regulation in these two growth stages. In addition, some of these propionylated sites are also acetylation and succinylation targets. For example, the propionylated site H3K57 is also modified by acetylation and succinylation, suggesting the concurrent roles of these PTMs in epigenetic regulation.

Histone PTMs have been shown to control many important biological processes, including DNA replication, DNA repair, gene regulation and accurate genome organization (Leach and Brown, 2012). Some studies have suggested that histone PTMs also contribute to fungal pathogenicity (Leach and Brown, 2012); however, histone propionylation has still not been extensively studied. In our study, only two propionylated sites (H3K57 and H4K13) were identified as conserved in other species, and 8 other histone sites have not been reported in the Compendium of Protein Lysine Modifications (CPLM) database and Protein Lysine Modifications Database (PLMD) thus far (Liu Z. et al., 2014; Xu H. et al., 2017). These 8 novel propionylated sites expand our knowledge of PTMs on histones. The specific roles of propionylation and the cooperation of propionylation functions with those of other PTMs in epigenetic regulation require further investigation.

### Conservation Analysis of the Propionylated Lysines

The conservation of propionylated lysines could be an indicator of the effect of protein propionylation. In our study, the propionylated and unpropionylated lysines in *T. ruburm* was compared to 10 species to investigate their evolutionary conservation. The selected species included *Homo sapiens*, *Mus musculus*, *Escherichia coli*, and seven fungus species. The results indicate that propionylated lysine is relatively more conserved than unpropionylated lysine (Supplementary Figure S4). The same phenomenon was also found for acetylated and malonylated lysines, which are also more conserved than unmodified lysine (Mo et al., 2015; Fan et al., 2017). Whether a stronger selective pressure exists to maintain the modified lysines during evolution or these PTMs tend to occur in more structurally conserved regions should be further investigated.

### Cross Talk Between the Propionylated Proteins and Acetylation- and Succinylation-Modified Proteins

In our previous study, 5,580 acetylated sites on 2,422 proteins and 569 succinylated sites on 284 proteins were identified in *T. rubrum* (Xu X. et al., 2017; Xu et al., 2018b). A comparison of the propionylome with the acetylome and succinylome revealed 77 propionylated proteins that were simultaneously modified by acetylation. Among these proteins, 42 proteins were modified at the same 55 lysine site by these two PTMs. In addition, 46 proteins were commonly modified by propionylation and succinylation, and 31 of these proteins were modified at the same 38 lysine site by these two PTMs. A total of 41 proteins were commonly modified by all three PTMs, and 25 sites on 20 proteins were commonly modified by all three PTMs (Supplementary Table S7).

**FIGURE 6:**
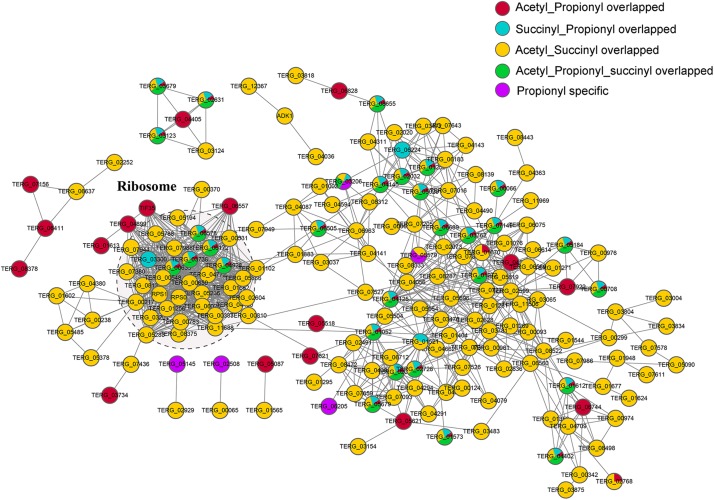
PPI networks of the propionylated, acetylated and succinylated proteins identified in *T. rubrum*. The red node represents the proteins commonly modified by Kac and Kpr; the blue node represents the proteins commonly modified by Ksucc and Kpr; the yellow node represents the proteins commonly modified by Kac and Ksucc; the green node represents the proteins commonly modified by Kac, Ksucc and Kpr; the purple node represents proteins specifically modified by Kpr.

To investigate the relationships of these different kinds of lysine-modification and their specific and cooperative roles in posttranslational regulation, we constructed protein-protein interaction (PPI) networks (Figure 6). The proteins were classified into five groups, including the proteins overlapping Kac and Kpr; proteins overlapping Ksucc and Kpr; proteins overlapping Kac and Ksucc; proteins overlapping Kac, Ksucc and Kpr; and Kpr-specific proteins. The most significantly enriched cluster was the ribosome, composed of proteins commonly modified by two or three of these PTMs, suggesting that ribosomal proteins are heavily modified by these lysine modifications.

### Relationship Between Propionylation Level and Protein Abundance

As shown in Figure 1, most propionylated proteins and sites are growth-stage specific in the conidial and mycelial stages. In our previous study, we found that proteins are largely differentially expressed between these two stages (Xu et al., 2018b). To investigate whether the difference in propionylation between conidia and mycelia was due to the difference in protein expression levels, we compared the propionylome and the whole-cell proteome between the two growth stages. Of the 45 proteins that are specific propionylated in the conidial stage, 3 were specific to the conidial stage in the whole-cell proteome, which may be due to the specific expression of these 3 proteins. Additionally, 10 of the 45 proteins that are specific propionylated in the conidial stage were more abundant in the conidial stage than in the mycelial stage, and 12 of the 45 proteins that are specific propionylated in the conidial stage were more abundant in the mycelial stage than in the conidial stage, based on the whole-cell proteome data. Of the 61 proteins that are specific propionylated in the mycelial stage, 2 were specifically expressed in the mycelial stage, 33 proteins were more abundant in the mycelial stage than in the conidial stage, and 2 proteins were more abundant in the conidial stage than in the mycelial stage, based on the whole-cell proteome data. For the conidia-specific propionylation modification, there was no preference for the proteins that were more abundant in conidia. For the mycelia-specific propionylated proteins, more than half of the proteins were more abundant in the mycelial stage, which may be explained by the results of the whole-cell proteome indicating that the proteins upregulated in the mycelial/conidial stage (1,612 proteins) were more abundant than the proteins downregulated in the mycelial/conidial stage (267 proteins). Based on these results, we conclude that there may be a slight bias of propionylation occurring on the proteins that are relatively more abundant in each stage, but there was no significant correlation between propionylation and protein abundance.

### The Propionyl-CoA Concentration Affects the Propionylation Level

**FIGURE 7:**
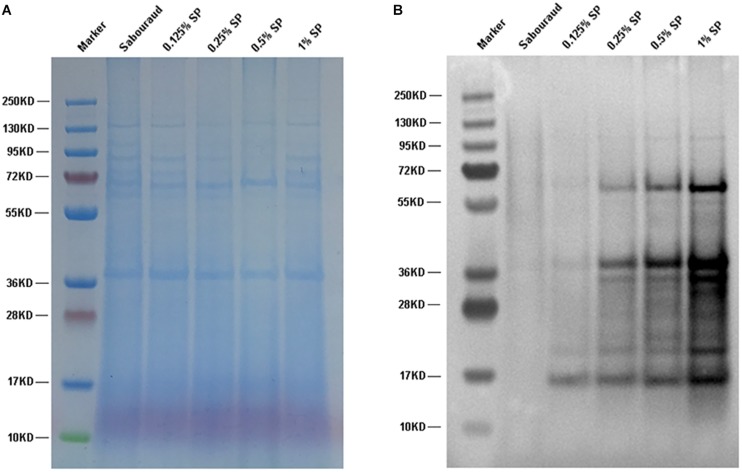
Western blot assay of *T. rubrum* cultured in different concentrations of sodium propionate to investigate the propionylation level. Sabouraud represents Sabouraud liquid medium with no addition of sodium propionate. SP represents sodium propionate that was supplemented to the Sabouraud liquid medium with different concentrations. **(A)** The loading control to ensure that equal amounts of protein were loaded in each lane. **(B)** Western blot results.

**FIGURE 8:**
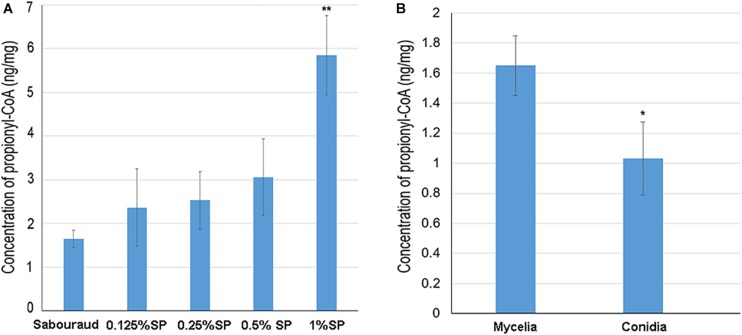
The intracellular propionyl-CoA concentrations of *T. rubrum*. Each bar represents the mean ± SD from three replicates. **(A)** The propionyl-CoA concentrations of *T. rubrum* that were cultured in different concentrations of sodium propionate (0.125, 0.25, 0.5, and 1% sequentially). Sabouraud represents Sabouraud liquid medium with no addition of sodium propionate. SP represents sodium propionate that was supplemented to the Sabouraud liquid medium with different concentrations. **(B)** The propionyl-CoA concentrations of *T. rubrum* in conidial and mycelial stages. The conidia and mycelia were cultured in potato dextrose agar and Sabouraud liquid medium respectively, with no addition of sodium propionate. Significant difference was calculated using *t*-test (^∗^ indicates *P* < 0.05, ^∗∗^ indicates *P* < 0.01).

Propionyl-CoA is the substrate for the Kpr reaction. To investigate whether the propionylation level is affected by propionyl-CoA concentration *in vivo*, *T. rubrum* was cultured in Sabouraud liquid medium and treated with different concentrations of sodium propionate (0.125, 0.25, 0.5, and 1% sequentially), which could be converted to propionyl-CoA (Sun et al., 2016; Han et al., 2018). As shown in Figure 7, the propionylated proteins were few in Sabouraud liquid medium, but the propionylation level increased for both histone and non-histone proteins in a concentration-dependent manner with the addition of sodium propionate. We further detected the intracellular propionyl-CoA concentrations of *T. rubrum*. The results show that the propionyl-CoA concentration increased with the increasing concentration of sodium propionate in the medium (Figure 8A and Supplementary Table S8). These results suggest that Kpr is affected by the substrate concentration of propionyl-CoA in *T. rubrum*.

The propionylation level in the mycelial stage is higher than in the conidial stage. We also tested the propionyl-CoA concentration in conidia to investigate whether difference exist between conidial and mycelial stages. As shown in Figure 8B, the propionyl-CoA concentration in mycelia cultured in Sabouraud is about 1.5 fold higher than in conidia. This different substrate concentration may contribute to the difference of propionylation abundance between conidial and mycelial stages.

### Effect on Fungal Viability When Cultured in Sodium Propionate

To investigate whether the growth of *T. ruburm* is affected by the addition of sodium propionate. We used XTT assay to determine fungal viability when cultured in different concentration of sodium propionate. As shown in Supplementary Figure S5, the cell viability is slight decreased when cultured in medium supplemented with 0.125 and 0.25% sodium propionate, but significantly decreased with the addition of 0.5 and 1% sodium propionate. These results show a phenomenon that addition of sodium propionate increased the propionylation level but inhibited fungal viability. Further experiments are needed to investigate whether the inhibition of fungal growth in sodium propionate is due to the increased propionylation level, and search for the reasons caused this growth inhibition.

### Discovery of Global PTMs in the *T. rubrum* Proteome

Currently, more than 620 kinds of PTMs have been experimentally discovered (Xu H. et al., 2018). In *T. rubrum*, except propionylation studied here, and two kinds of PTMs, acetylation and succinylation, have been identified in previous studies (Xu X. et al., 2017; Xu et al., 2018b); whether other types of PTMs exist in this fungus is still unknown. In this study, we adopted the combined unrestrictive and specific database search strategy illustrated by Yang et al. (2018) to search for possible PTMs in *T. rubrum* using data from the in-depth whole-cell proteome identification of *T. rubrum* performed previously (Xu et al., 2018b). We assigned 26 common modification types in eukaryotes for database searching, including ubiquitination (C, S, T, and K), phosphorylation (S, T, and Y), methylation (E, K, and R), crotonylation (K), and butyrylation (K), to search for their existence in this fungus. As a result, all 26 types of PTMs were identified in the *T. rubrum* proteome. A total of 5,778 unique peptides with various PTMs were identified on 2,205 proteins (Supplementary Table S2B).

To validate the existence of these PTMs in *T. rubrum*, we performed a western blot assay using the modification-specific antibodies. Seven kinds of PTM-specific antibodies, anti-butyrylation (K), anti-dimethylation (K), anti-trimethylation (K), anti-2-hydroxyiso-butyrylation (K), anti-malonylation (K), anti-ubiquitylation (K), and anti-glutarylation (K), were used in the western blot assay. All seven tested PTMs were identified in *T. rubrum*, validating the search results (Supplementary Figure S6). For several kinds of PTMs, such as butyrylation, dimethylation and trimethylation, the modification abundance shows a significant difference between conidia and mycelia, suggesting that these PTMs are differentially involved in these two growth stages.

## Discussion

In our study, we performed a proteome-wide analysis of Kpr in *T. rubrum*. A total of 156 propionylated sites were identified on 115 proteins, accounting for 1.1% of the *T. rubrum* proteome. When compared the propionylation level with other organisms, we found that the percent for propionylated protein is 1.6% in *Geobacillus kaustophilus*; 8% in *T. thermophilus*; 9.5% in *E. coli*; 0.1% in *Bacillus subtilis* and 0.07% in *Rhodothermus marinus* (Okanishi et al., 2014, 2017; Sun et al., 2016). The propionylation level is distinct in these kinds of organisms, but in general it is a kind of low abundant PTM as we known. The PRM assay was performed using two synthetic propionylated peptides, showing high confidence of the propionylation identification. The propionylated proteins were mostly involved in various metabolic processes, suggesting their major roles in regulating metabolism. Some pathogenicity-related proteins were also found to be propionylated. In addition, 10 propionylated sites were identified on histones. In particular, 8 sites on histones have not been reported in other species thus far, which expanded our knowledge of histone propionylation modification.

A comparison of the propionylation in the conidial and mycelial stages revealed that most propionylated proteins and sites were specific to each growth stage. To elucidate whether these great differences in propionylation modification were due to differences in protein abundance, the propionylomes were compared to the whole-cell proteome. The results show that propionylation modification was not significantly associated with protein abundance. Thus, we speculate that the propionylation level is regulated independently of protein abundance. When detecting the propionyl-CoA concentration in the conidial and mycelial stages, the results show more abundant propionyl-CoA in mycelia than in conidia, which may contribute to the different propionylation level between the two stages. Based on GO and KEGG classification, the propionylated proteins participate in some similar biological processes between the two stages and are specifically involved in some roles in a growth-stage-specific manner.

The comparison of the propionylome results with the acetylome and succinylome of *T. rubrum* indicated that these three acylations displayed different modification levels. Acetylation most widely exists and is much more abundant in mycelia than in conidia (Xu et al., 2018b). However, succinylation and propionylation have significantly lower modification levels than acetylation and show similar abundances in conidia and mycelia (Xu X. et al., 2017). Based on the functional analysis, these three PTMs show a similar GO distribution. Metabolism was the largest category for all three PTMs, indicating that these PTMs are all greatly involved in metabolism in *T. rubrum*. In addition, a number of pathogenicity-related proteins have been found to be modified by these three kinds of PTMs, and 9 proteins are commonly modified by these PTMs. Furthermore, all three PTMs are specifically and commonly modified on histones. The specific roles of each PTM and their cooperative participation in various cellular processes require further exploration.

In our previous study, we found that disturbed acetylation status significantly inhibited fungal growth (Xu et al., 2018b). In the present study, when cultured with the addition of sodium propionate, the propionylation level in *T. ruburm* significantly increased, and the growth of this fungus was significantly inhibited. Although the relations of propionylation level and fungal viability is need further investigations, this results may provide some potential clues for the anti-fungal researches.

The propionyl group has only one carbon atom more than the acetyl group (Sabari et al., 2017). Many studies focused on the enzymes catalyzing these two PTMs (Smith and Denu, 2007; Cheng et al., 2009). The balance of each kind of PTM is regulated by two types of enzymes: acyltransferases and deacylases (Sabari et al., 2017). For instance, acetylation is controlled by KATs and KDACs (Albaugh et al., 2011). KATs add the acetyl group to the lysine residue, which can be classified into three major families: Moz, Ybf2, Sas2 and Tip60 (MYST); Gcn5-related *N*-acetyltransferases (GNATs); and CREB-binding proteins (p300/CBP) (Sabari et al., 2017). Due to the structural similarity of acetyl-CoA to the short-chain acyl-CoAs, it has been suggested that acetyltransferases possess promiscuous acyltransferase activity (Sabari et al., 2017). In a recent study, it was validated that all three KAT families possess KPT activity (Han et al., 2018). In contrast to KATs, KDACs catalyze the removal of acetylation modification (Smith and Denu, 2007). KDACs could be classified as NAD^+^-dependent sirtuin deacetylases (SIRT1-7) and Zn^2+^-dependent histone deacetylases (HDAC) (Hyndman and Knepper, 2017). Some KDACs also possess the activities to catalyze the removal of Kpr (Sabari et al., 2017). In our study, the amino sequence motif surrounding the lysine sites was different between acetylation and propionylation, which is an indicator of the sequence character recognized by the enzyme catalyzing each type of PTM (Figure 2) (Xu et al., 2018b). This poses the question of whether differences exist between the enzyme identifying and catalyzing these two kinds of PTMs. The specific mechanisms of these enzymes that catalyze each kind of PTM, whether propionylation-specific enzymes exist, and the specificity of these enzymes in *T. rubrum* require further investigation.

In addition, although there is structural similarity between acetylation and propionylation, the abundances of these two PTMs are largely different, and propionylation is much less abundant than acetylation. This difference may be due to the following possible reasons. First, different efficiencies of acyltransferases that catalyze the addition of acetyl and propionyl residues may exist. Second, differences may also exist in the efficiencies of the deacylases that catalyze the removal of the acetyl and propionyl residues. For example, Hst2 is a KDAC in yeast, which has a greater affinity for binding propionyl-lysine than acetyl-lysine (Albaugh et al., 2011). Third, there may be a difference in the abundances of the substrates for these two kinds of PTMs. Acyl-CoA and propionyl-CoA are donors of these two PTMs, respectively (Albaugh et al., 2011). It has been shown in human embryonic kidney 293T (HEK293T) cells that the abundance of propionyl-CoA is eight times less than that of acetyl-CoA (Han et al., 2018). Furthermore, our results show that increasing the concentration of propionyl-CoA significantly increased the propionylation level in *T. rubrum*. This result suggests that the abundance of propionyl-CoA is also an essential factor impacting the propionylation level.

In addition to propionylation, acetylation and succinylation identified in *T. rubrum*, we questioned whether other types of PTMs also exist in this fungus. In our study, we used a combined unrestrictive and specific database search strategy to identify various PTMs in *T. rubrum*. All 26 assigned PTM types were identified in the *T. rubrum* proteome, and 7 of these PTM types were selected and validated by western blot. All of these PTMs are involved in posttranslational regulation in *T. rubrum* and may work together to participate in various biological processes. Further studies of various PTMs in *T. rubrum* could improve our understanding of the biological roles of these PTMs and provide the foundation for the research of regulatory mechanisms for these types of PTMs.

## Conclusion

To our knowledge, our study is the first proteome-wide propionylation analysis in *T. rubrum* and in a pathogenic fungus. This study provides an analysis of propionylation modifications and their roles in various important biological pathways in *T. rubrum*. The results also reveal the properties of propionylation in each of the two major growth stages of this fungus and the differences in propionylation between the two major growth stages. Our study provides a foundation for further research on the roles of PTMs in posttranslational regulation in *T. rubrum*, which will enhance our understanding of the physiology of *T. rubrum* and facilitate the identification of improved therapies to treat this medically important fungi.

## Data Availability Statement

The datasets generated for this study can be found in the Peptide Atlas, PASS01412, https://db.systemsbiology.net/sbeams/cgi/PeptideAtlas/PASS_View.

## Author Contributions

XX and XC performed the experiments and wrote the manuscript. JY, LC, and BL performed the bioinformatics analysis. QJ and TL designed the research and reviewed the manuscript. All authors read and approved the submitted version.

## Conflict of Interest

The authors declare that the research was conducted in the absence of any commercial or financial relationships that could be construed as a potential conflict of interest.

## Footnotes

1 http://www.broadinstitute.org/annotation/genome/dermatophyte_comparative/MultiDownloads.html

2 http://www.ebi.ac.uk/GOA/

3 https://skyline.ms/project/home/software/Skyline/begin.view

4 https://www.ncbi.nlm.nih.gov/Taxonomy/Browser/wwwtax.cgi?id=559305

## Abbreviations

DDA: data-dependent acquisition
DIA: data-independent acquisitio
FDR: false discovery rate
GO: Gene Ontology
KAT: lysine acetyltransferase
KDAC: lysine deacetylases
KEGG: Kyoto Encyclopedia of Genes and Genomes
Kpr: lysine propionylation
KPT: lysine propionyltransferase
LC-MS/MS: liquid chromatography-tandem mass spectrometry
PPIs: protein–protein interactions
PRM: parallel reaction monitoring
PTM: post-translational modification
T. rubrum: Trichophyton rubrum
