# Supplementary material for: Proteome-Wide Identification of Lysine Propionylation in the Conidial and Mycelial Stages of Trichophyton rubrum
Source: Front Microbiol. 2019 Nov 13;10:2613. doi: 10.3389/fmicb.2019.02613 (PMC6861857; doi:10.3389/fmicb.2019.02613)
Supplement: Supplementary file 1 [file Data_Sheet_1.PDF]

Supplementary Figures

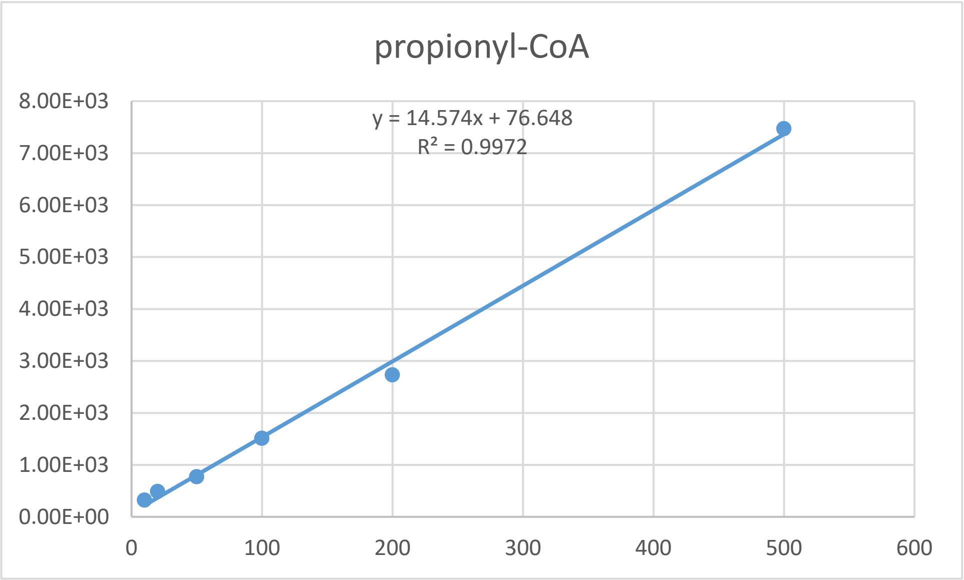

Supplementary Figure S1. The standard curve of propionyl-CoA. The abscissa indicates the concentration of standard propionyl-CoA (ng/ml), and the ordinate indicates the peak area.

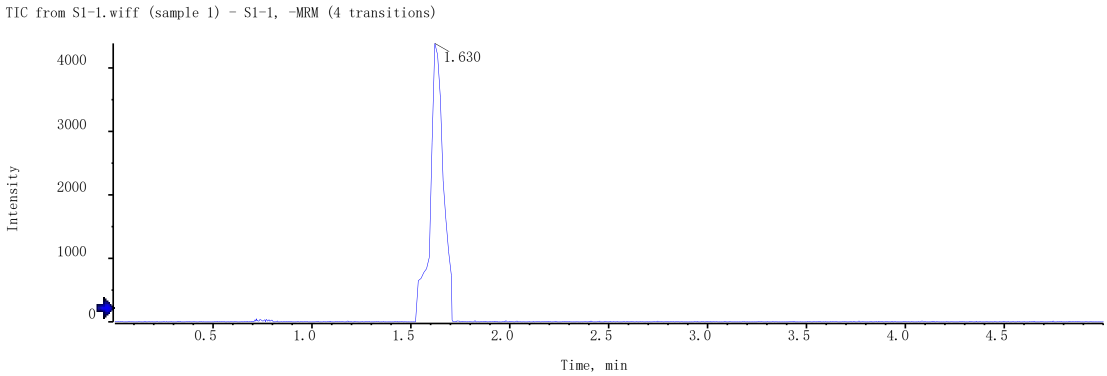

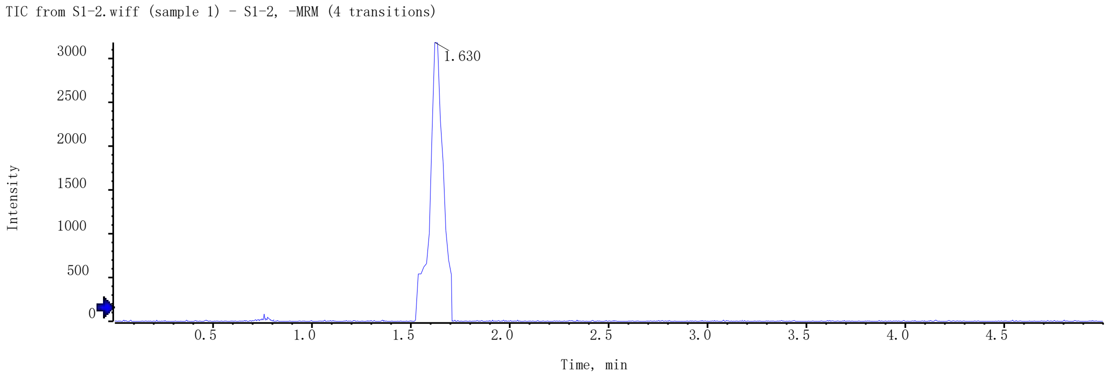

TIC from S1-3.wiff (sample 1) - S1-3, -MRM (4 transitions)

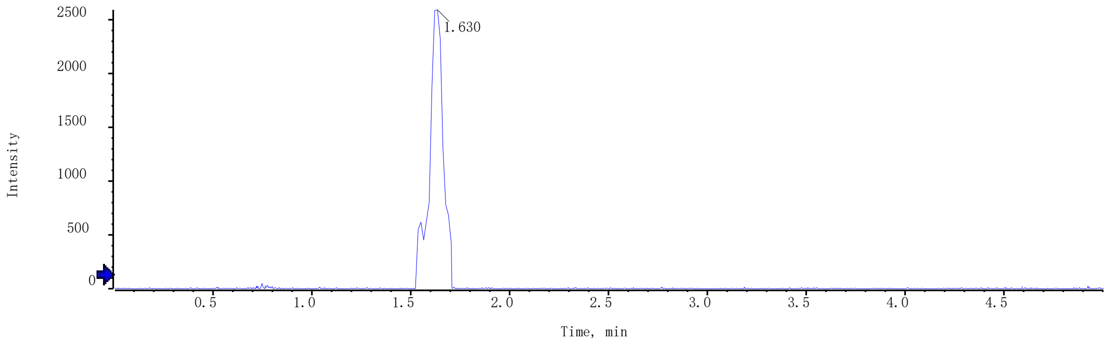

TIC from S2-1.wiff (sample 1) - S2-1, -MRM (4 transitions)

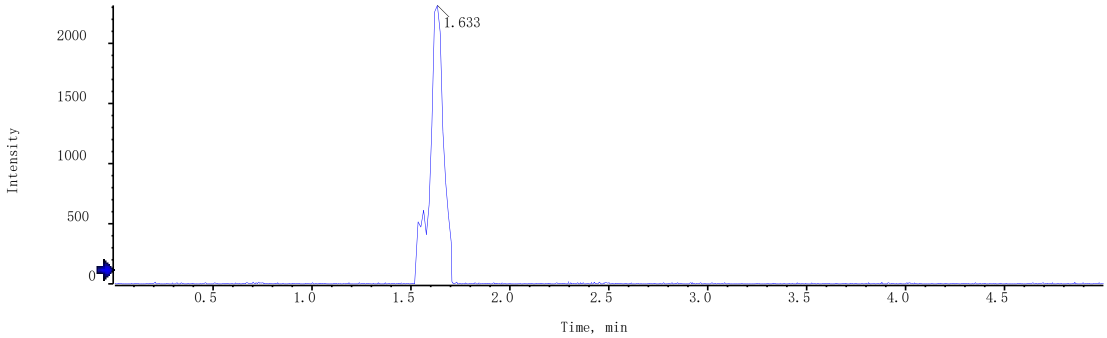

TIC from S2-2.wiff (sample 1) - S2-2, -MRM (4 transitions)

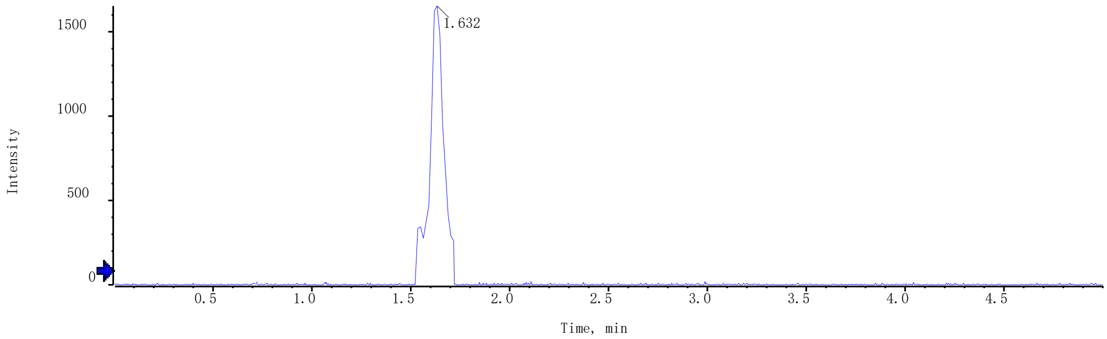

TIC from S2-3.wiff (sample 1) - S2-3, -MRM (4 transitions)

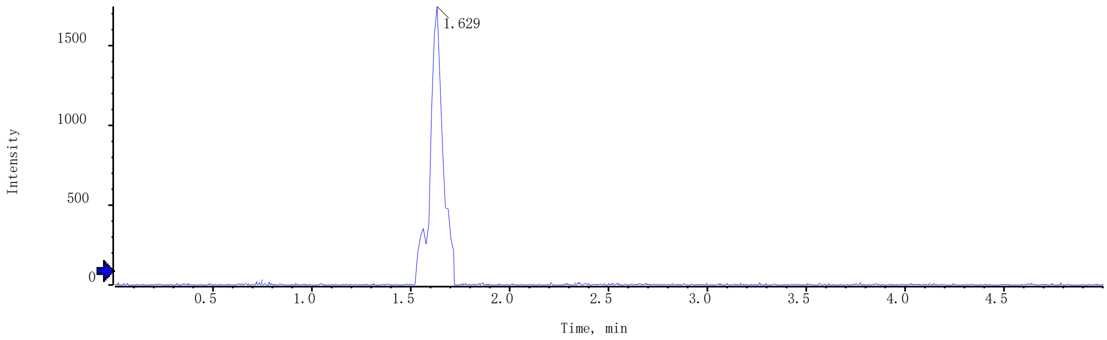

TIC from S3-1.wiff (sample 1) - S3-1, -MRM (4 transitions)

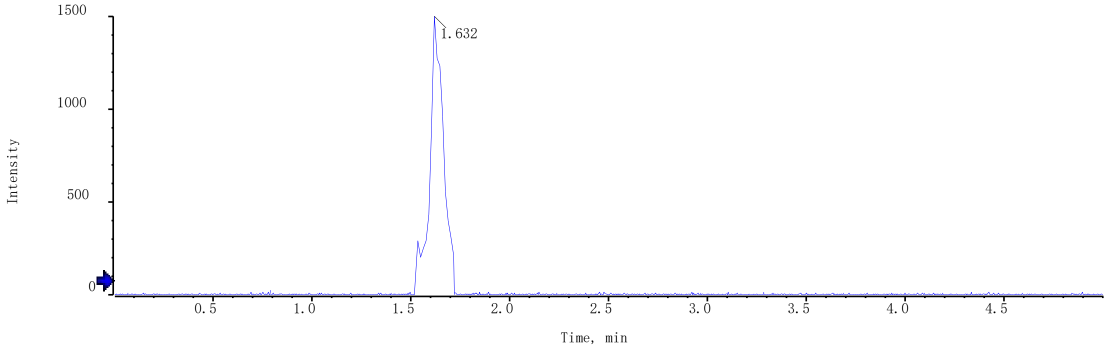

TIC from S3-2.wiff (sample 1) - S3-2, -MRM (4 transitions)

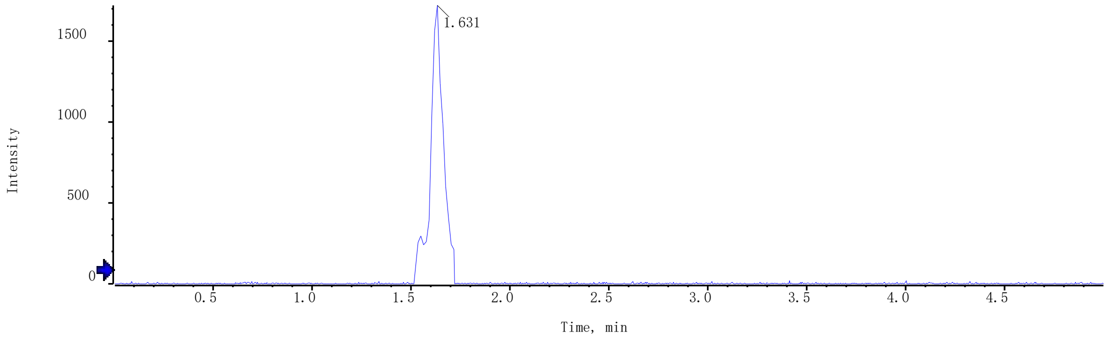

TIC from S3-3.wiff (sample 1) - S3-3, -MRM (4 transitions)

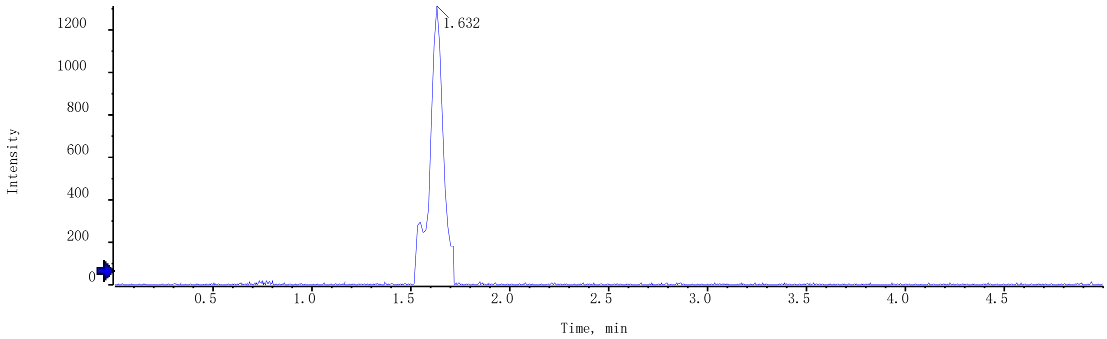

TIC from S4-1.wiff (sample 1) - S4-1, -MRM (4 transitions)

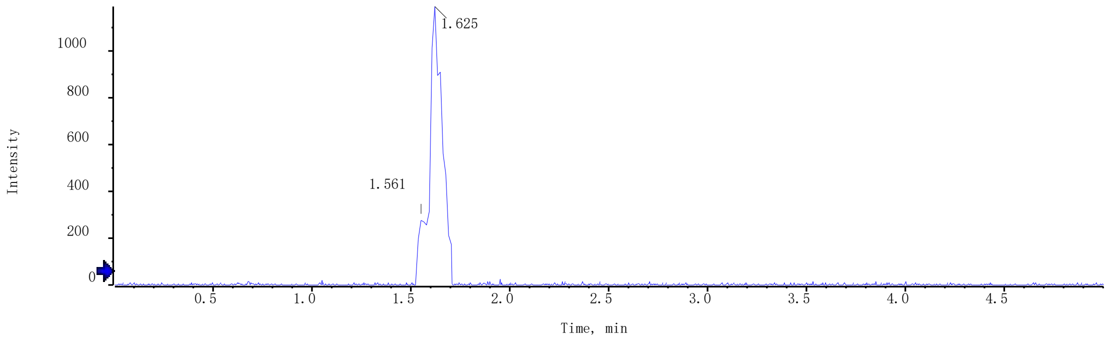

TIC from S4-2.wiff (sample 1) - S4-2, -MRM (4 transitions)

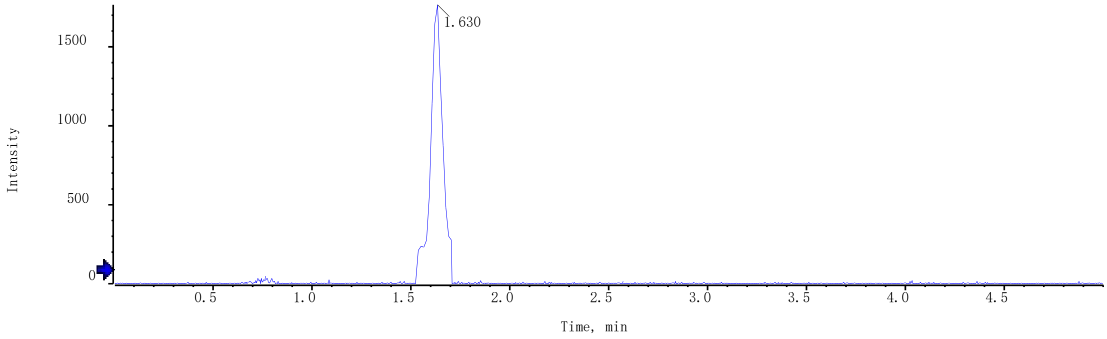

TIC from S4-3.wiff (sample 1) - S4-3, -MRM (4 transitions)

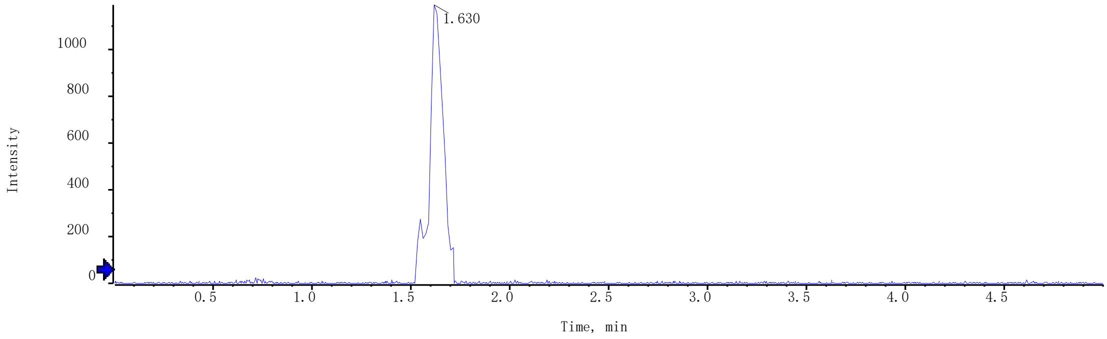

TIC from S5-1.wiff (sample 1) - S5-1, -MRM (4 transitions)

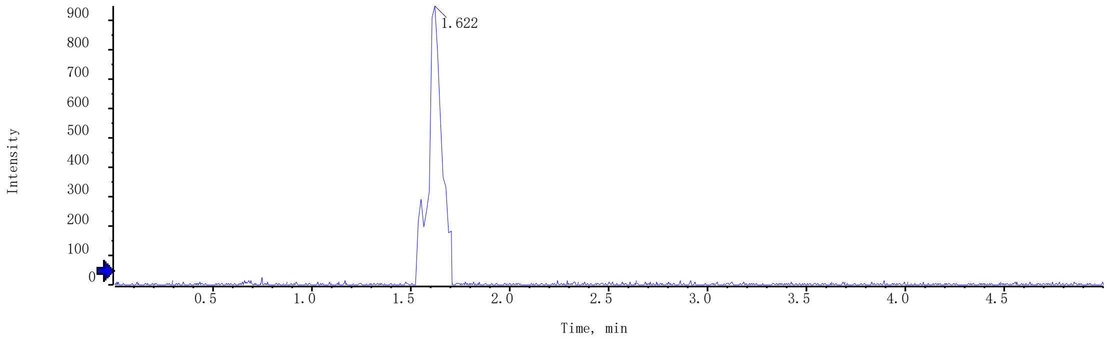

TIC from S5-2.wiff (sample 1) - S5-2, -MRM (4 transitions)

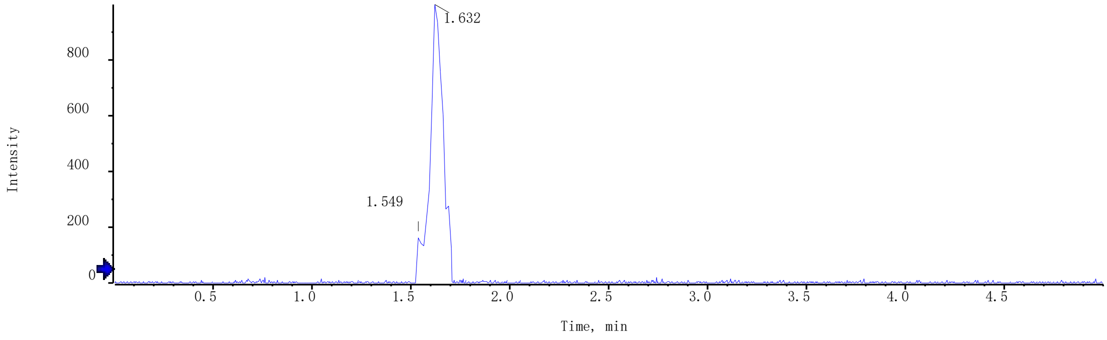

TIC from S5-3.wiff (sample 1) - S5-3, -MRM (4 transitions)

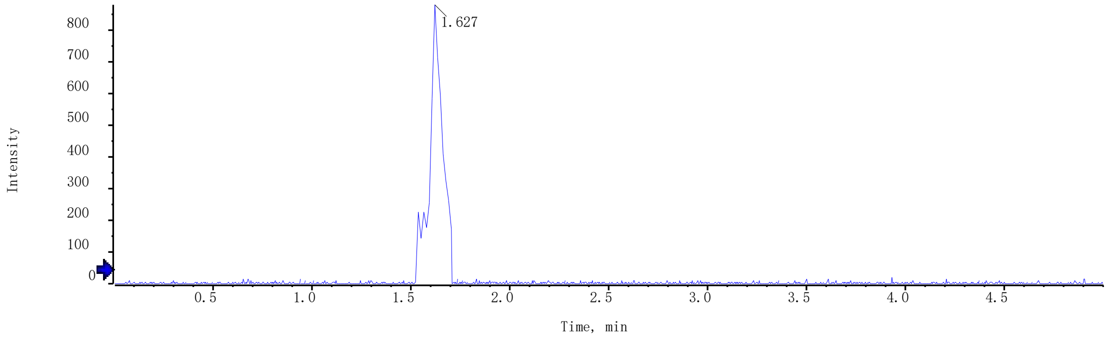

TIC from S6-1.wiff (sample 1) - S6-1, -MRM (4 transitions)

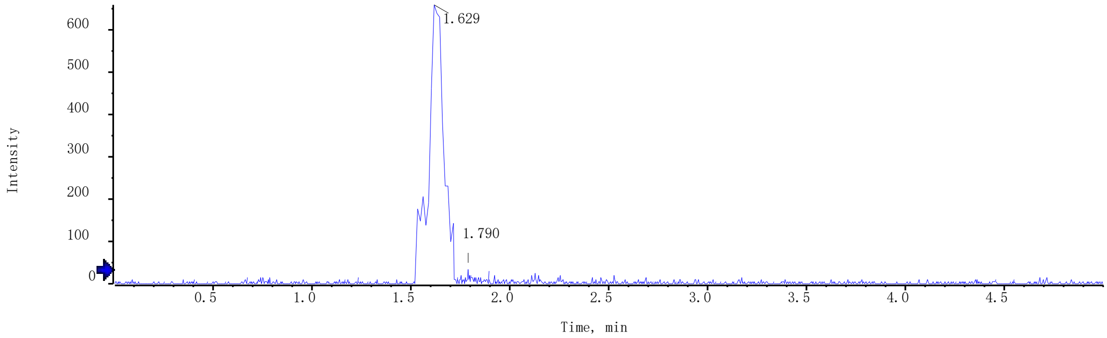

TIC from S6-2.wiff (sample 1) - S6-2, -MRM (4 transitions)

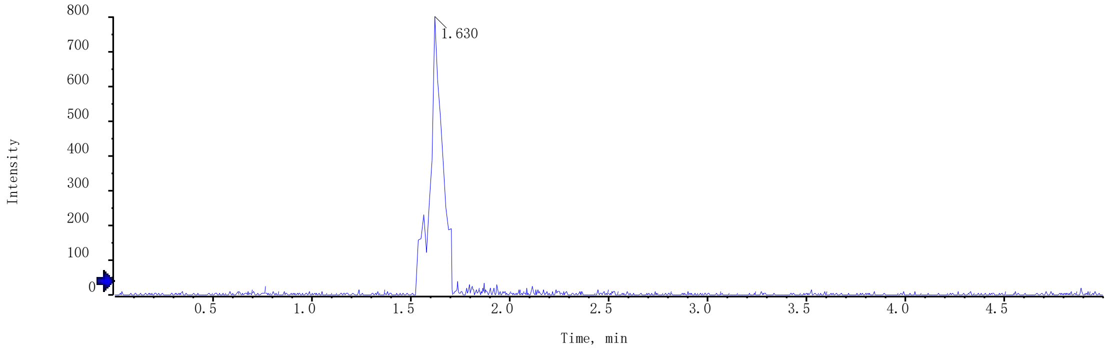

TIC from S6-3.wiff (sample 1) - S6-3, -MRM (4 transitions)

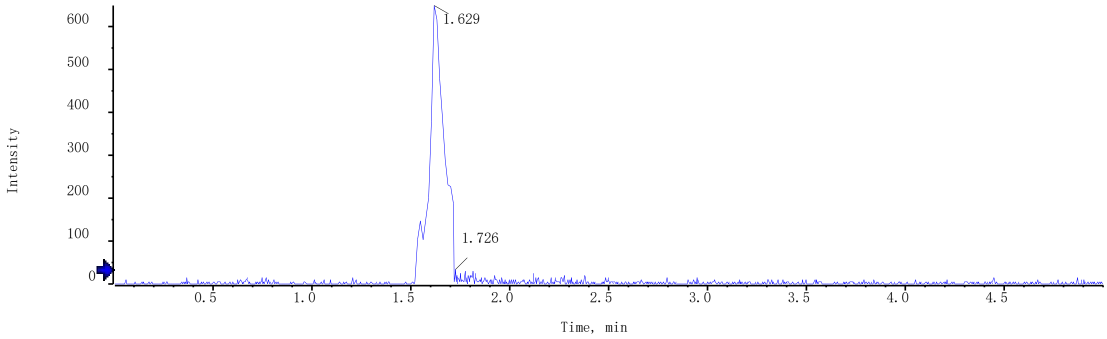

TIC from std-10.wiff (sample 1) - std-10, -MRM (4 transitions)

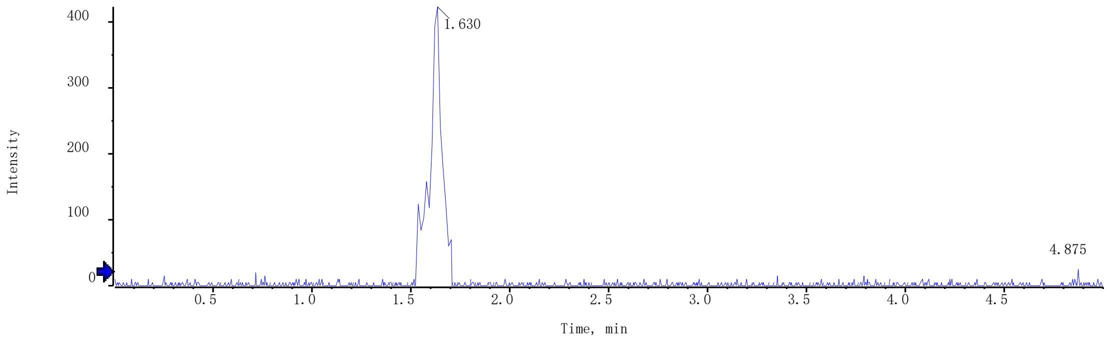

TIC from std-20.wiff (sample 1) - std-20, -MRM (4 transitions)

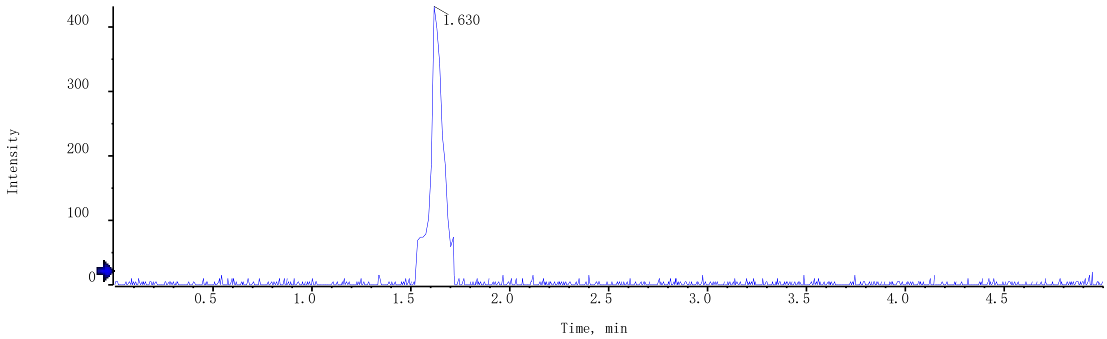

TIC from std-50.wiff (sample 1) - std-50, -MRM (4 transitions)

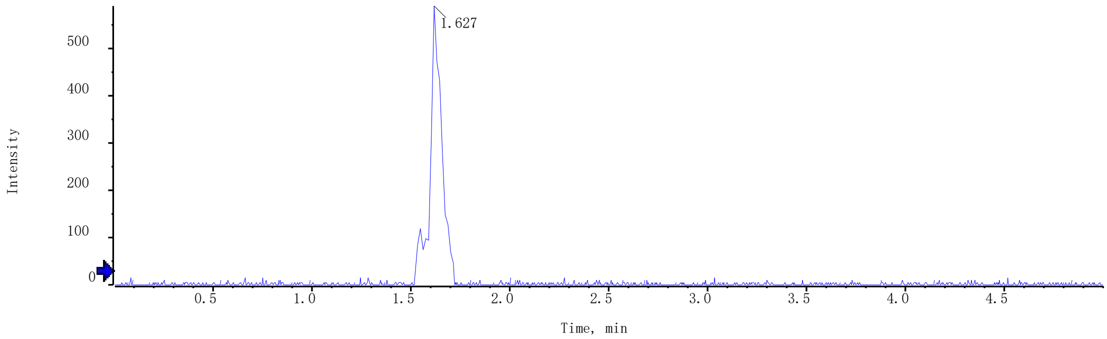

TIC from std-100.wiff (sample 1) - std-100, -MRM (4 transitions)

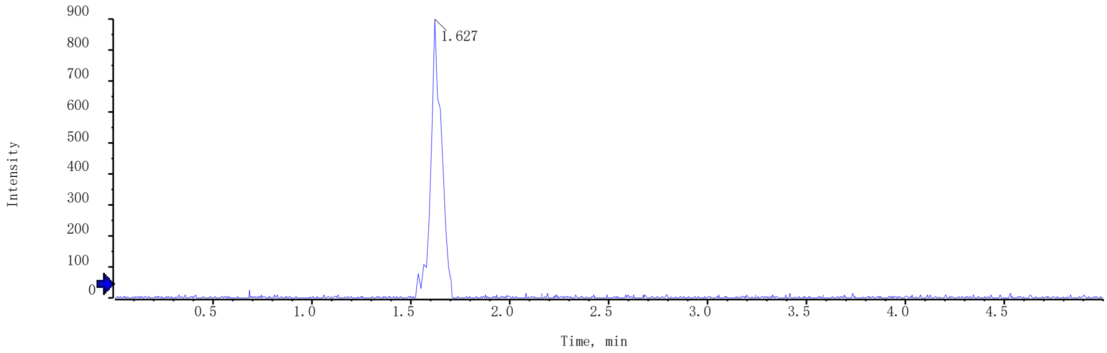

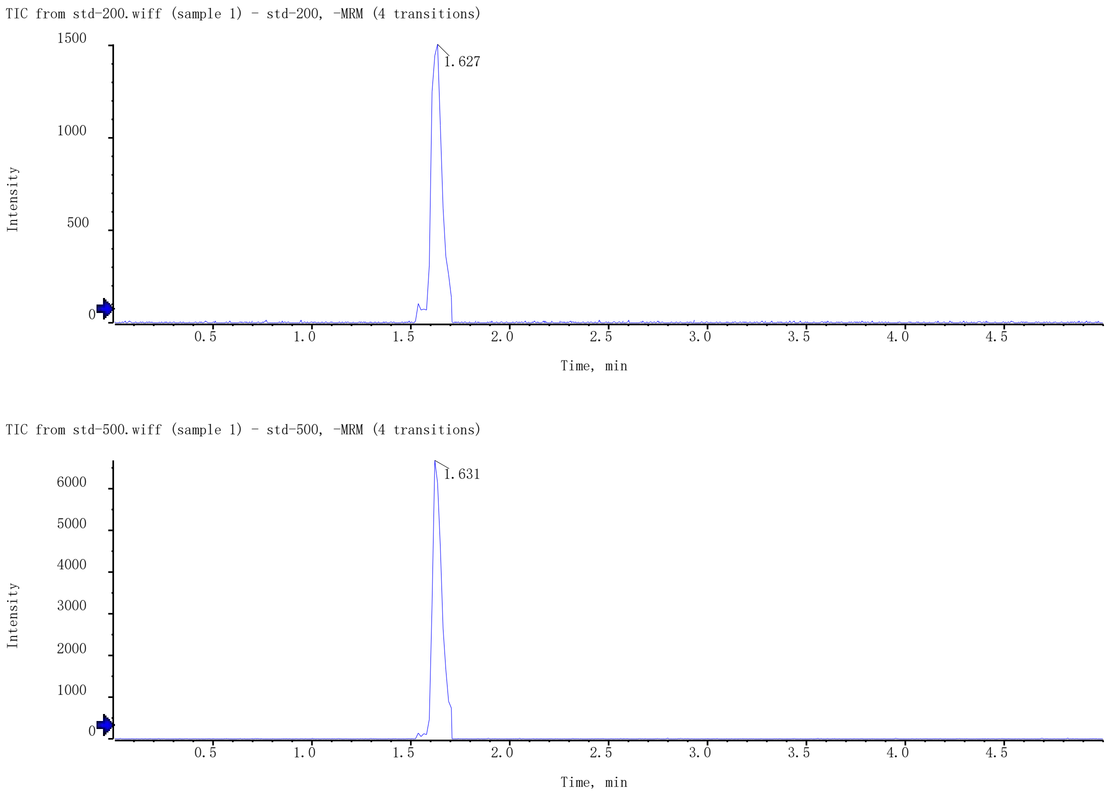

Supplementary Figure S2. The total ion current (TIC) and the retention time for each sample.

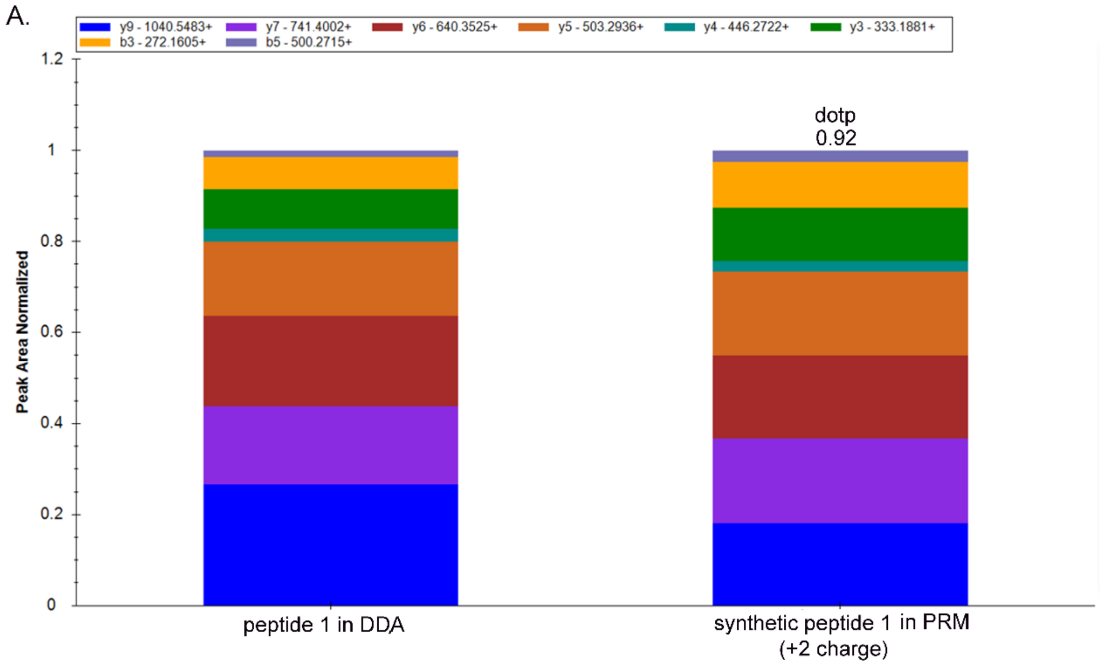

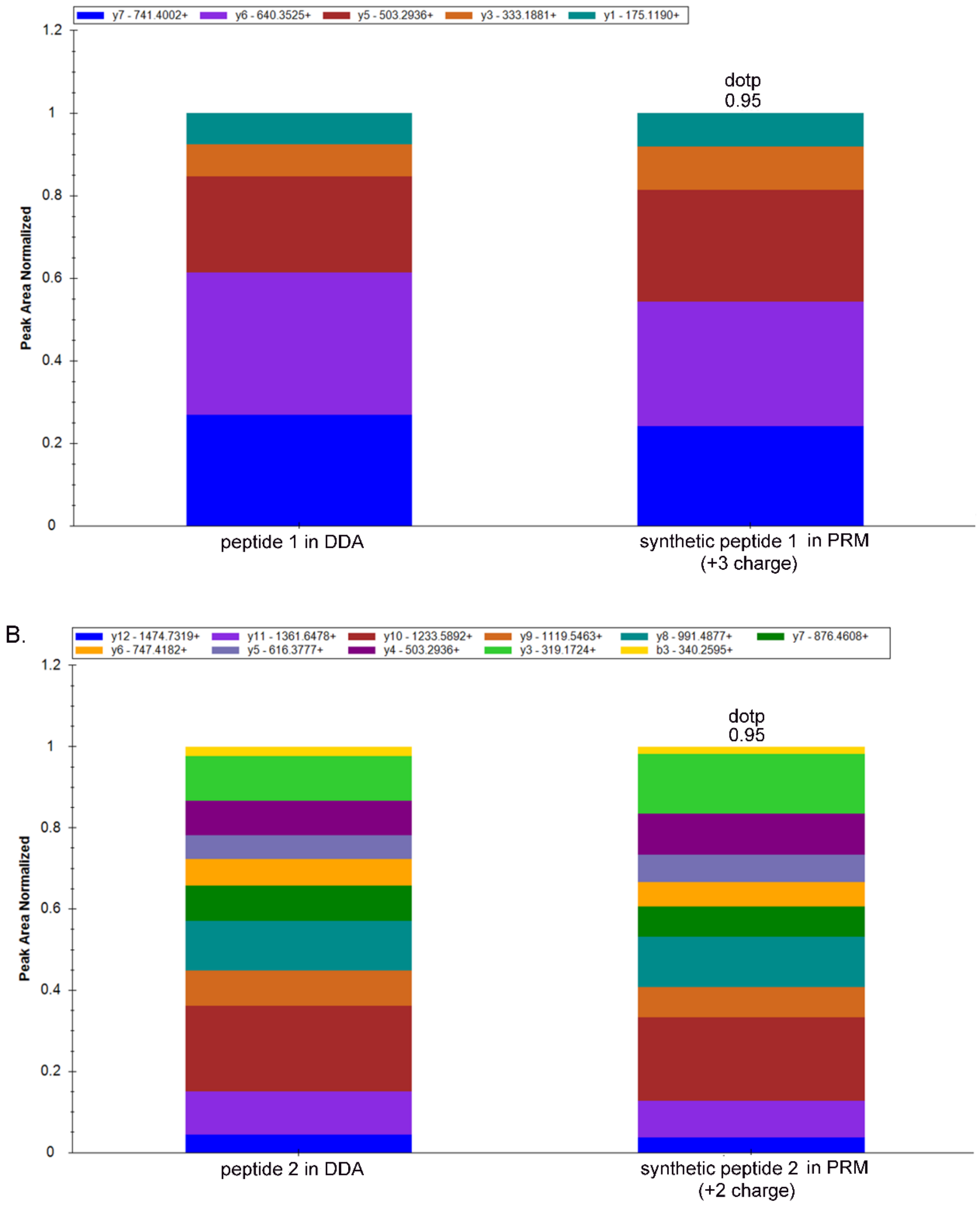

Supplementary Figure S3. PRM validation of the propionylated peptide identification. The spectra of the synthetic peptides identified in the PRM mode were compared to the specific peptides identified in the DDA mode in the initial propionylome analysis. The “dotp” value illustrates the similarity of the fragment ion intensity between the synthetic peptide and their corresponding peptides. Two synthetic propionylated peptides were used for PRM validation. **(A)** Synthetic peptide 1 (AISLDK(pro)THGISAR), including +2 charged fragment ions and +3 charged fragment ions respectively. **(B)** Synthetic peptide 2 (LLIQNQDEMLK(pro)SGR), including +2 charged fragment ions only.

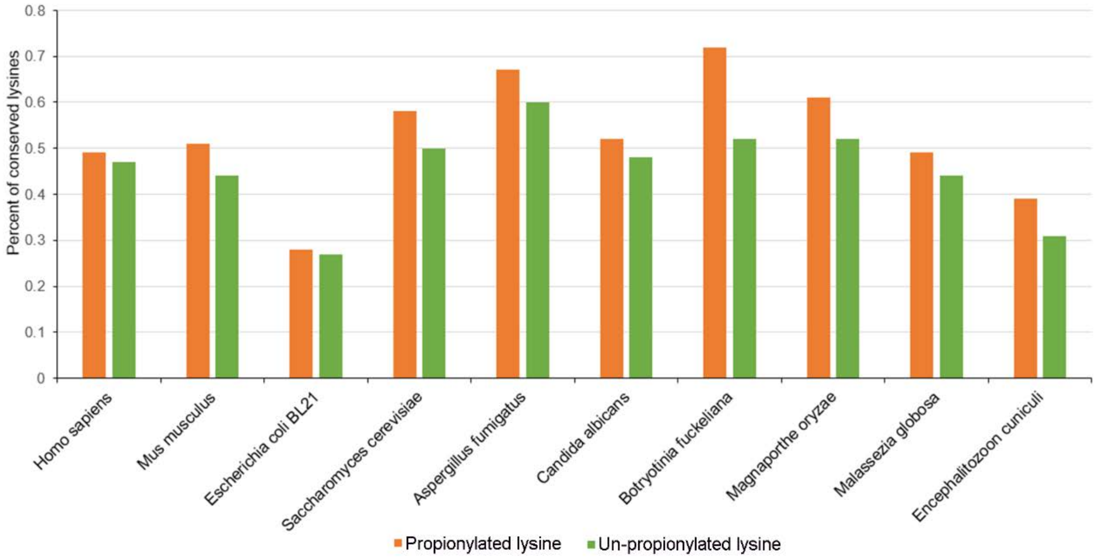

Supplementary Figure S4. Conservation of propionylated and un- propionylated lysine in *T. rubrum* compared with other species.

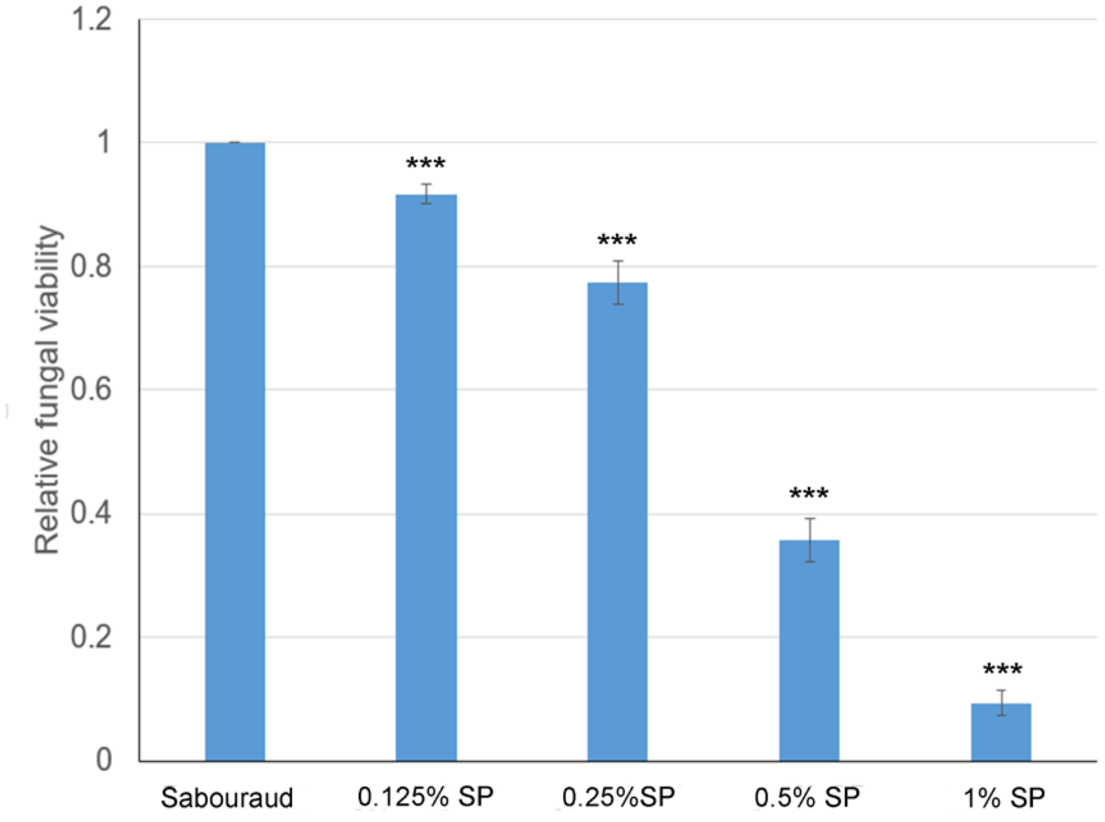

Supplementary Figure S5. The relative viability of *T. rubrum* cultured in Sabouraud liquid medium compared to the addition of different concentration of sodium propionate (SP). Three independent replicates were performed. Significant difference was calculated using t-test.

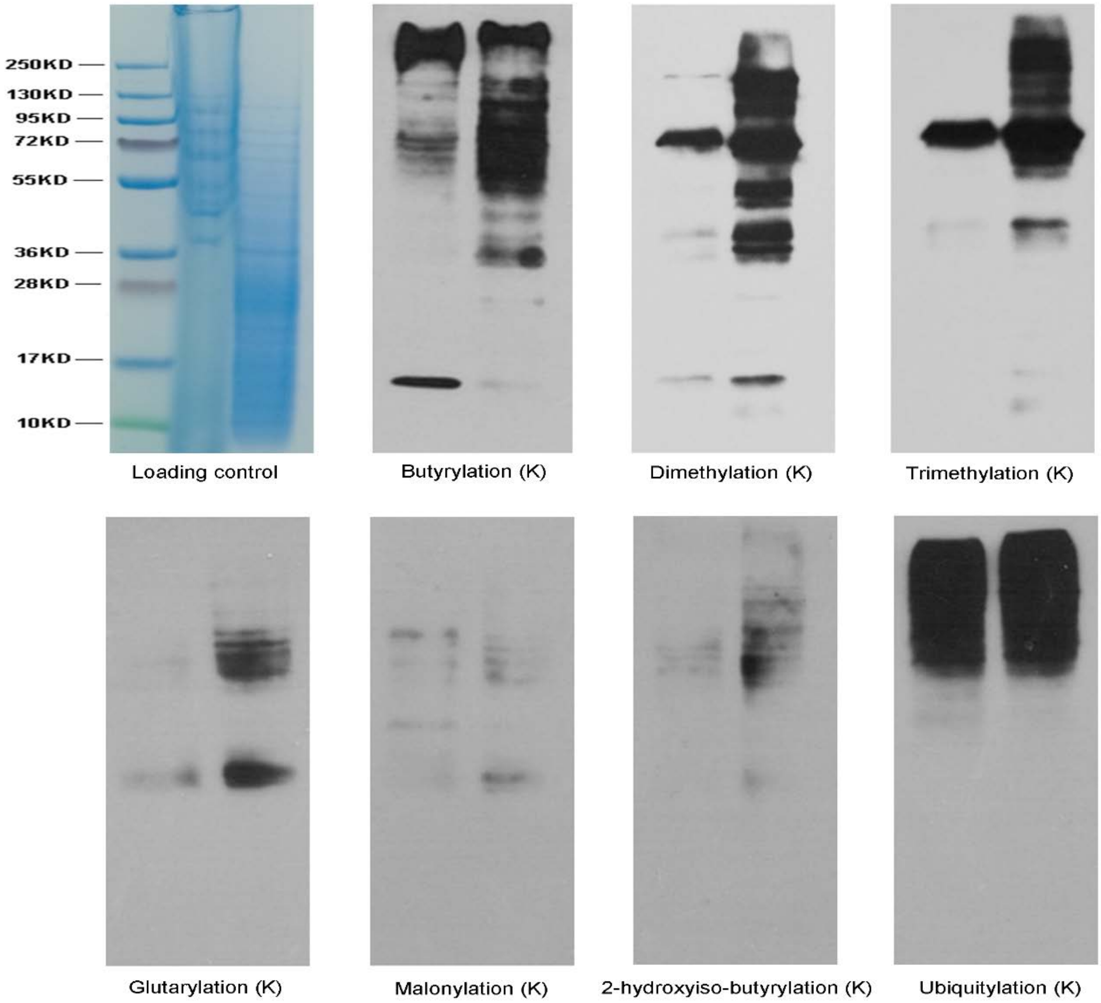

Supplementary Figure S6. Validation the existence of PTMs in *T. rubrum* using modification-specific antibodies. The left lane represents the conidial stage, and the right lane represents the mycelial stage.
